# Synthesis of well-defined linear–bottlebrush–linear triblock copolymer towards architecturally-tunable soft materials†

**DOI:** 10.1039/d2py00841f

**Published:** 2022-07-20

**Authors:** Vahid Asadi, Xuecong Li, Francesco Simone Ruggeri, Han Zuilhof, Jasper van der Gucht, Thomas E. Kodger

**Affiliations:** Physical Chemistry and Soft Matter, Wageningen University & Research Stippeneng 4 6708 WE Wageningen The Netherlands thomas.kodger@wur.nl; Laboratory of Organic Chemistry, Wageningen University & Research Stippeneng 4 6708 WE Wageningen The Netherlands; Department of Chemical and Materials Engineering, Faculty of Engineering, King Abdulaziz University 21589 Jeddah Saudi Arabia

## Abstract

Linear–bottlebrush–linear (LBBL) triblock copolymers are emerging systems for topologically-tunable elastic materials. In this paper, a new synthetic methodology is presented to synthesize LBBL polystyrene-*block*-bottlebrushpolydimethylsiloxane-*block*-polystyrene (PS-*b*-bbPDMS-*b*-PS) triblock copolymer *via* the “grafting onto” approach where the precursors are individually synthesized through living anionic polymerization and selective coupling reaction. In this two-step approach, polystyrene-*block*-polymethylvinylsiloxane (PS-*b*-PMVS) diblock copolymer with a low dispersity couples with another living PS block to form PS-*b*-PMVS-*b*-PS triblock copolymer. Secondly, this is followed by grafting of separately prepared monohydride-terminated PDMS chains with controllable grafting density through a hydrosilylation reaction. In addition to fully tunable architectural parameters, this approach permits a quantitative determination of the ratio of diblock and triblock bottlebrush copolymers and consistency between batches, highlighting the feasibility for scaled-up production. These LBBL triblock copolymers self-assemble into soft, low-modulus thermoplastic elastomers, and the precise knowledge of the composition is crucial for correlating microstructure to mechanical properties.

## Abstract 2

A new method is discussed to synthesize linear–bottlebrush–linear polystyrene-*block*-polydimethylsiloxane-*block*-polystyrene triblock copolymer *via* “grafting onto” approach with well-defined and tunable architectural parameters.

## Graphical abstract

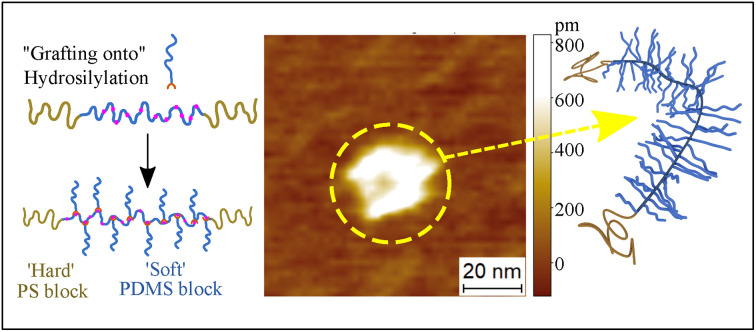

## 1 Introduction

Block copolymers, composed of two or more chemically distinct polymer chains, can self-assemble into ordered nanostructures driven by the inherent immiscibility of the differing polymer segments. This facilitates the use of block copolymers in many technologically important applications, including lithography templates, porous structures for filtration and separation, and drug carriers.^1–5^ Among different configurations of block copolymers, ABA triblock copolymers are an important subset that microphase separate to physically connected elastic networks known as thermoplastic elastomers. These are used in everyday items requiring a low modulus, such as soft touch toothbrush grips, to more industrial uses in medical devices, in the construction industry, and in many other advanced systems.^6^ One example of this class of materials is polystyrene-*block*-polydimethylsiloxane-*block*-polystyrene (PS-*b*-PDMS-*b*-PS) where the middle PDMS block with a low glass transition temperature (*T*_g_) provides elastomeric properties at room temperature, while the PS block with a high *T*_g_ value acts as a physical crosslinker.^7^ Apart from its exceptionally low *T*_g_ and high flexibility, PDMS features low reactivity, low surface energy, low absorption in UV, high biocompatibility and thermal stability, all of which render PDMS-based ABA copolymers ideal for designing low-modulus, soft materials.^8,9^

Chemically immiscible block copolymers undergo phase separation of mesoscopic dimensions defined by the molecular stoichiometry established during their synthesis, which in turn drives their mechanical properties; this has inspired polymer chemists to precisely program the molecular architecture of each block toward the final desired material properties. However, linear block copolymers also often contain entanglements that serve as additional effective crosslinks, and hence limit their function by increasing the moduli. For certain applications, such as tissue engineering, soft materials with moduli approximately that of biological tissues are needed while entanglements often limit polymeric material to a higher moduli, *i.e. E* ≈ 200 kPa for PDMS, and thus limit their potential usage in applications requiring lower moduli.^10^ One promising way to prevent entanglements is utilizing an alternative polymeric topology, a “bottlebrush” polymer, where a polymer backbone has been highly grafted with relatively short side chains. These side chains effectively play the role of solvent by diluting backbones which lower the modulus by reducing the number density of phase separated domains in thermoplastic elastomers without addition of any solvent or additives. In addition to backbone dilution, the grafted side chains face steric repulsion between side chain neighbors leading to backbone stretching which significantly increases the entanglement molecular weight.^11–13^ Recently, there is a growing research interest in linear–bottlebrush–linear (LBBL) triblock copolymers to create ultrasoft and even strain-stiffening thermoplastic elastomers which closely mimic the mechanical response of biological tissue.^14–16^ However, the synthesis of LBBL copolymers in a controlled and consistent way is beset with challenges.

Combining PDMS properties with the LBBL molecular topology would yield such a ultrasoft material where the tunability is enhanced with precise topology and low dispersity. PDMS bottlebrush (BB) architectures have been highlighted in the literature through several synthetic approaches, each with distinctive advantages. Such topological polymers can be made by quenching anionically synthesized living PDMS chains with a chlorine-functionalized backbone which is prepared using equilibrium polymerization followed by a chlorination step.^17,18^ However, this approach leads to higher *Đ* values due to the inherent nature of equilibrium polymerization, and hence requires multiple fractionation steps. PDMS bottlebrushes have also been synthesized using the highly functional “grafting through” approach where PDMS macromonomers link together from a single polymerizable end group. In this case, PDMS macromonomers are either end-functionalized with norbornene units which are able to link together through ring opening metathesis polymerization (ROMP)^19,20^ or with (meth)acrylate units suitable for controlled radical polymerization such as ATRP.^21,22^ Interestingly, this latter approach also enables the synthesis of a series of PDMS-based LBBL triblock topology with chemically different polymers as linear blocks.^14,23,24^ An inherent advantage of “grafting through” is the highest grafting density as each backbone unit possesses one side chain. Additionally, “grafting-through” possesses a low concentration of polymerizable end groups and steric hindrance of the propagating chain end, both reducing the macromonomer conversion and propagation rate.^25^ Unfortunately, chain-breaking events such as biradical termination or chain transfer remain a challenge when employing ATRP for “grafting through” polymerization of macromonomers.^26^ This results in undesired linear–bottlebrush diblocks (LB) and bottlebrush homopolymer (BB) impurities of an unknown quantity which can alter bulk properties such as elasticity and relaxation dynamics.^23^ Consequently, networks formed of such LBBL triblocks with the same targeted chemical and architectural composition have a noticeable batch-to-batch difference in mechanical properties.^23^ Additionally, PDMS-based bottlebrushes synthesized with this “grafting through” approach possess a backbone chemistry that is different from the PDMS side chains which affects the network mechanical properties.^27^ Therefore, developing a protocol that minimizes impurities while also precisely controlling the molecular chemistry and architecture would ensure a high consistency in mechanical properties of a topologically driven soft material.

In this paper, we discuss an alternative methodology for preparing LBBL PS-*b*-bbPDMS-*b*-PS triblock copolymer using a “grafting onto” approach where the precursors, *i.e.* side chains and backbone, are individually synthesized through anionic polymerization and subsequently selectively coupled. Although this “grafting onto” approach suffers from limited grafting density due to steric repulsion between side chains, the mechanical response of most biological soft tissues can be well-mimicked by bottlebrush networks in the lower range of grafting densities. Separate formation of precursors with a very low dispersity, *Đ*, employing anionic polymerization^28^ allows for their characterization individually to enable high level of precision on the molecular structure. Additionally, we use anionic polymerization, an industrial method producing yearly kilograms of polymers possibly enabling scale-up as the necessary industrial equipment and expertise exist. This approach technically features a few highlights: numerous lengths of side chains and grafting densities can be made with an identical batch of backbone leading to high consistency in the final properties of LBBL triblocks; quantitatively determining the ratio of diblock and triblock in the final material giving better insight about the mechanical properties; and suitability for scale-up and mass production as challenging purification steps are not required.

## 2 Experimental

### 2.1 Materials

All chemicals were used as received unless otherwise noted. *n*-Butyllithium (*n*-BuLi, 2 M in cyclohexane), *sec*-butyllithium (*sec*-BuLi, 1.4 M in cyclohexane), tetrahydrofuran (THF, anhydrous, ≥99.9%, inhibitor-free), styrene, platinum(0)-1,3-divinyl-1,1,3,3-tetramethyldisiloxane complex solution (in xylene, Pt ≈ 2%), 1,1-diphenylethylene (DPE, 97%), lithium bromide (LiBr, ≥99%), and chlorotrimethylsilane (purified by redistillation, ≥99%) were purchased from Sigma-Aldrich. Hexamethylcyclotrisiloxane (D_3_, 95%), 1,3,5-trivinyl-1,3,5-trimethylcyclotrisiloxane (D_3_V), chlorodimethylsilane (CDMS, 98%), and ((chloromethyl)phenylethyl)dimethylchlorosilane (CMPDMS) were purchased from Gelest. Anhydrous toluene was obtained from Acros Organics and other solvents such as cyclohexane, methanol, and acetone were purchased from Biosolve.

### 2.2 Instrumentation

Size exclusion chromatography (SEC) experiments were conducted on an Agilent system equipped with differential refractometer using one Agilent organic column (PLgel MIXED-D, 7.5 × 300 mm, 5 μm) with THF or toluene as eluent at 35 °C and a flow rate of 1 mL min^−1^. Molecular weights and molecular weight distributions (*M*_w_/*M*_n_) were measured relative to linear polystyrene standards. Nuclear magnetic resonance (^1^H NMR) spectra were recorded using a Bruker 400 MHz instrument at 25 °C with deuterated chloroform (99.50% D) as internal standard.

Sample deposition for atomic force microscopy (AFM) analysis was performed on two differently charged substrates: hydrophobic highly ordered pyrolytic graphite (HOPG) and negatively charged mica. Sample preparation was realized at room temperature by deposition of a 40 μL droplet of 10 μM fabricated PS-*b*-bbPDMS-*b*-PS triblock copolymer solution in THF for 40 seconds in the case of mica and deposition of a 10 μL droplet for 40 seconds in the case of HOPG. After the incubation time on the surface, the sample was rinsed with 1 mL Milli-Q water and then dried by the passage of a gentle flow of gaseous nitrogen. Phase-controlled and high-resolution AFM imaging was realized by a MultiMode VIII Scanning Probe Microscope (Bruker, USA), operating in tapping mode and equipped with an antimony n-doped Si tip (Veeco, TAP150A, 5 nm^−1^) with a nominal radius of 7 nm. Image flattening and analysis was performed by SPIP (Image metrology) software.^29–31^

Rheological experiments were performed on a stress-controlled rheometer (TA, Discovery Hybrid) with parallel plate geometry with diameter of 8 mm at a gap size of 850 μm. The sample was loaded on the bottom plate and melted at 140 °C, shown in Fig. S14,† followed by squeezing and trimming of excess sample. After equilibrating for a period of 10 min at 140 °C to erase the loading history, an oscillatory temperature sweep measurement was done from 140 °C to 20 °C at rate of 2 °C min^−1^, 5% strain, and frequency of 1 Hz. The stress–relaxation experiments were done with a step strain of 5% at 140 °C, 80 °C, and 20 °C over a period that ensures stable stress value or complete relaxation to zero stress. The sample was allowed to equilibrate at respective temperatures for a period of 20 min to ensure consistent network formation conditions. Frequency sweep response of the sample was captured from 10^−4^ to 10^1^ Hz with strain of 5% at 20 °C, shown in Fig. S15.†

Differential scanning calorimetry (DSC) measurements were performed using DSC250 (TA Instruments) in nitrogen atmosphere. First, the sample of approximately 6 mg weight was equilibrated at −85 °C for 1 minute followed by heating at rate of 10 °C min^−1^ up to 150 °C, held for 1 minute to produce a uniform thermal history. Then, the sample was cooled down to −85 °C with rate of 10 °C min^−1^, equilibrated for 1 minute and reheated back to 150 °C at the same ramp of 10 °C min^−1^. The thermal stability of the synthesized samples was analyzed by thermogravimetric analysis (TGA) using Mettler Toledo TGA/DSC 1 at a heating rate of 5 °C min^−1^ from room temperature to 800 °C under inert atmosphere on samples with an average mass of 6 mg. Results can be seen in the ESI in Fig. S16 and S17.†

### 2.3 Synthesis procedures

All glassware was baked at 110 °C overnight and immediately transferred to a nitrogen environment glove box for all anionic polymerizations. All reactions were carried out at room temperature unless otherwise stated. D_3_ monomer was purified by sublimation on a Schlenk line. Activated molecular sieves (4 Angstrom, Sigma-Aldrich) were used to dry cyclohexane for at least 48 hours before usage. Styrene was purified by vacuum distillation at 40 °C and stored at −80 °C in the presence of activated molecular sieves.

#### 2.3.1 Monohydride-terminated polydimethylsiloxane (H-PDMS)

Sublimed D_3_ (50.0 g, 224.76 mmol) was dissolved in anhydrous cyclohexane (400.82 mL) followed by the addition of *n*-BuLi (5.62 mL, 11.24 mmol) as initiator. After overnight reaction at room temperature, anhydrous THF (44.53 mL) was added to the reaction medium as a promoter to reach a solvent composition of cyclohexane/THF (90/10) and subsequently, began chain propagation. The reaction proceeded for 20 hours to reach 50% conversion, determined by ^1^H NMR spectroscopy. The reaction was quenched by the addition of 2 molar equivalents of CDMS (2.5 mL, 22.47 mmol). After the reaction mixture was allowed to stand overnight to fully quench the reaction, it was filtered, and the majority of the solvent was evaporated by a rotary evaporator. Unreacted monomers (D_3_) along with the remaining solvent were removed using a strong vacuum equipped with a vacuum trap.

#### 2.3.2 Synthesis of PS-*b*-PMVS-*b*-PS triblock copolymer

##### 2.3.2.1 Synthesis of PS-b-PMVS diblock copolymer

Distilled styrene (2.5 g, 24 mmol) was dissolved in anhydrous cyclohexane (80.24 mL) followed by the addition of *sec*-BuLi (343 μL, 0.48 mmol) to initiate its polymerization. After complete polymerization over 4 hours, D_3_ (427 mg, 1.92 mmol) was added to the reaction medium from a stock solution (187 mg mL^−1^) along with THF (834 μL). After overnight reaction, the orange color of the living polystyrene had disappeared, and more THF (8.30 mL) was added to change the solvent composition to cyclohexane/THF (90/10). At this point, D_3_V (6.41 mL, 24.0 mmol) was quickly injected into the reaction flask. Polymerization of D_3_V proceeded for 24 hours and was quenched with CMPDMS (136 μL, 0.55 mmol). The quenching step continued for another 24 hours followed by precipitation into methanol, and twice reprecipitation into methanol from a THF solution, resulting in BnCl-functionalized PS-*b*-PMVS with isolated weight yield of 95%.

##### 2.3.2.2 Halogen exchange of BnCl-functionalized PS-b-PMVS diblock copolymer

BnCl-functionalized PS-*b*-PMVS diblock copolymer (500 mg) along with dried LiBr (250 mg) were dissolved in 25 mL of acetone and *n*-heptane mixture with a 3/1 volume ratio. The resulting solution was purged with nitrogen for 10 minutes and refluxed overnight. The reaction mixture was then poured into methanol. The product was further purified by reprecipitation from THF solution into methanol and dried in a vacuum oven overnight. The halogen exchange reaction that forms BnBr-functionalized PS-*b*-PMVS diblock copolymer was followed by ^1^H NMR spectroscopy as shown in Fig. S9.†

##### 2.3.2.3 Coupling reaction of BnBr-functionalized PS-b-PMVS diblock with another PS block

Another block of PS was separately prepared from a styrene (520 mg, 4.99 mmol) solution in cyclohexane (4.5 mL) initiated with *sec*-BuLi (71 μL, 0.1 mmol). After 4 hours of polymerization, THF was added to change the solvent composition to cyclohexane/THF (90/10) followed by end-capping with a 3 times molar excess of DPE (52.9 μL, 0.3 mmol) for an hour. The DPE end-capped living PS was poured into a THF solution of BnBr-functionalized PS-*b*-PMVS diblock (500 mg in 6.2 mL). The reaction mixture was allowed to stand for 3 days to form the desired triblock. After this time, 1.2 equiv. of chlorotrimethylsilane compared to DPE end-capped PS was used to terminate any living chains. The reaction mixture was poured into a large amount of methanol to precipitate the triblock, uncoupled diblock and inert PS.

#### 2.3.3 Synthesis of PS-*b*-bbPDMS-*b*-PS triblock copolymer

The product of the previous step (292 mg) and the synthesized H-PDMS (597 mg) were dissolved in anhydrous toluene (3 mL). This mixing ratio was determined based on the stoichiometric calculation of available vinyl groups per gram of the PS-*b*-PMVS-*b*-PS triblock mixture containing excess PS. At this stage, an aliquot from the solution was taken as a reference point for further characterization. Karstedt's catalyst (4.5 μL, 2% platinum in xylene), was added to reach a catalyst concentration of 5 μL per gram of undissolved material. The temperature was raised to 80 °C to increase the rate of reaction and the grafting reaction by hydrosilylation was monitored with ^1^H NMR spectroscopy. The reaction was stopped after 25% of vinyl groups had been consumed leading to 25% grafting density. After evaporation of solvent, the resulting material was dissolved in a mixture of acetone/THF (80/20) and precipitated into acetone/methanol (70/30) twice to remove excess PS along with unreacted H-PDMS.

## 3 Results and discussion

**Fig. 1:**
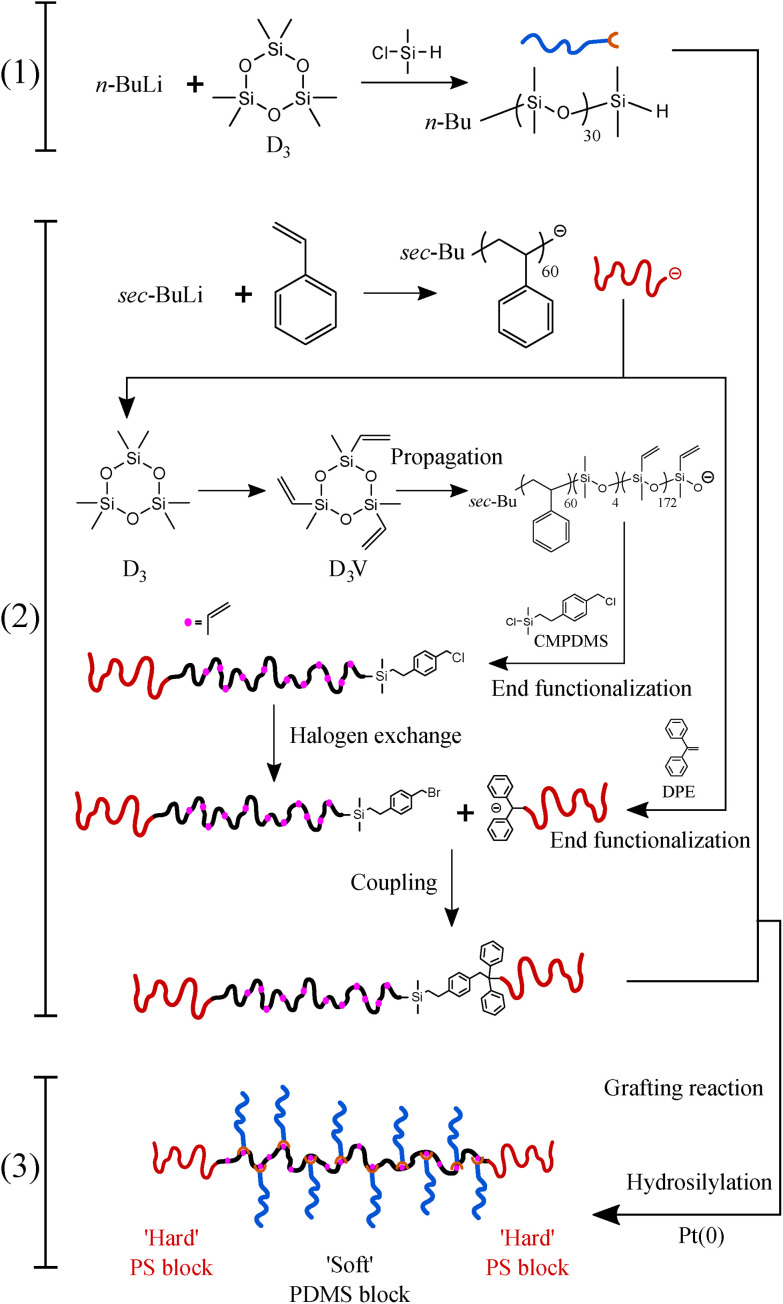
Controlled synthesis scheme of PS-*b*-bbPDMS-*b*-PS triblock copolymer. (1) Anionic polymerization of D_3_, (2) anionic polymerization of PS-*b*-PMVS diblock copolymer followed by coupling with another PS block to make PS-*b*-PMVS-*b*-PS triblock copolymer, (3) grafting of H-PDMS chains on the obtained triblock copolymer through hydrosilylation reaction to synthesis bottlebrush chains.

The LBBL molecular topology in triblock copolymers enables a synergy between microphase separation of the end blocks and the avoidance of entanglements due to the bottlebrush middle block to create elastically tunable physical networks. However, producing PDMS-based LBBL thermoplastic elastomers with consistent properties has been an ongoing challenge due to the intrinsic and hard-to-control complications during the commonly-used “grafting through” synthesis approach, such as termination and chain transfer events which lead to undesired linear–bottlebrush diblocks (LBB) and bottlebrush homopolymer (BB) impurities.^23^ Here, PS-*b*-bbPDMS-*b*-PS triblock copolymers are created by a sequence of steps classified in three main categories: (1) synthesis of monodispersed H-PDMS side chains, (2) synthesis of PS-*b*-PMVS-*b*-PS triblock copolymer backbone, followed by (3) the “grafting onto” of the PDMS side chains on the middle block of the triblock copolymer backbone, as shown in Fig. 1 with corresponding numbers. This approach allows for both the synthesis of high purity and low dispersity components and a quantification of the grafting density using ^1^H NMR.

### 3.1 Synthesis of the H-PDMS

**Fig. 2:**
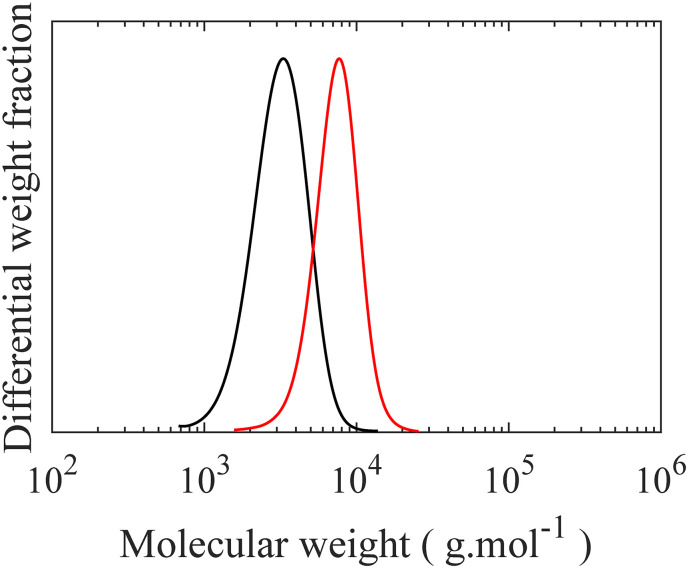
SEC curves of H-PDMS with two different degree of polymerization. (**—**) *M*_n_ = 2800, *M*_w_ = 3400 g mol^−1^, and *Đ* = 1.19, (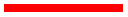) *M*_n_ = 6800, *M*_w_ = 7700 g mol^−1^, and *Đ* = 1.13.

Controlled synthesis of PDMS is typically performed by anionic ring-opening polymerization of D_3_, a cyclotrisiloxane with ring strain, initiated by organolithium initiators.^7,32,33^ First, the initiator reacts with D_3_ to form short silanolate ions which attack other D_3_ monomers. However, no polymerization occurs even in the presence of excess D_3_ unless a donor solvent, a promoter such as THF, is added to the reaction medium. Addition of the promoter disrupts the self-association of the end ions, permitting chain propagation. This restriction is highly advantageous to form low dispersity (*Đ*) polymers by increasing the initiation efficacy by separating it from chain propagation. A monohydride functional group, which serves as an anchor point in the grafting step, is introduced to the propagating end of the chain by near-quantitative quenching of the polymerization with excess chlorodimethylsilane (CDMS).^34^ A high degree of monofunctionality in H-PDMS is crucial, as difunctionality can lead to crosslinking reactions between molecular brushes during the final “grafting onto”, while non-functionalized side chains do not graft. The CDMS is added when the total amount of D_3_ polymerized is 50%. If polymerization continues to full conversion, a fraction of dihydride functionality occurs, which is only observable after grafting, making quantification challenging as shown in Fig. S1–S4.† Previous works have described side reactions at high conversion including backbiting and reshuffling processes, both chain scission and recombination, which may form cyclic compounds, broaden the molecular weight distribution, and possibly introduce difunctional chains.^35^ The degree of polymerization of the H-PDMS side chains is controlled by changing the initial ratio of monomer to initiator during the polymerization. SEC chromatographs of the resulting H-PDMS with low *Đ* for two different molecular weights, having DP of 10 and 21, are shown in Fig. 2, with additional kinetic information of H-PDMS polymerizations and ^1^H NMR spectra of obtained polymers being found in Fig. S5–S7.† Excellent hydride end functionalization is calculated to be around 99% for both molecular weights based on the ratio of –Si–H (4.70 ppm) to –CH_3_ (0.88 ppm) signals in ^1^H NMR spectra.

### 3.2 Synthesis of the linear PS-*b*-PMVS-*b*-PS triblock copolymer

**Fig. 3:**
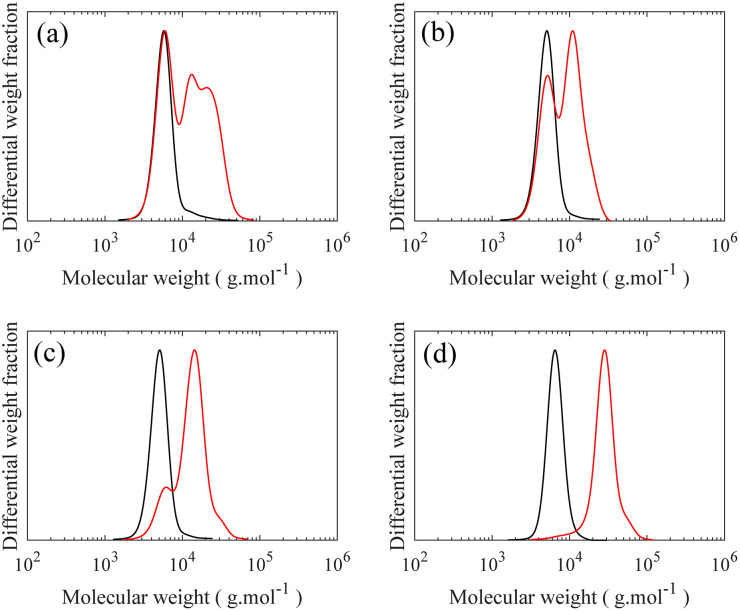
SEC curves of (**—**) PS and (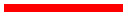) corresponding PS-*b*-PMVS diblock copolymer synthesized by sequential monomer addition following initial styrene polymerization: (a) a second monomer (D_3_V) was added and led to react with living PS overnight followed by 10 v% THF addition, (b) D_3_V was added along with 10 v% THF, (c) a few units of D_3_ per chain was added and led to react with living PS overnight followed by addition of D_3_V and 10 v% THF, (d) a few units of D_3_ per chain and 1 v% THF was added simultaneously and led to react with living PS overnight followed by addition of D_3_V and 10 v% THF. Note: (**—**) PS, *M*_n_ = 6300, *M*_w_ = 6700 g mol^−1^, *Đ* = 1.06, (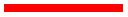) PS-*b*-PMVS diblock copolymer, *M*_n_ = 23 200, *M*_w_ = 27 100 g mol^−1^, *Đ* = 1.16.

To form a LBBL topology using the “grafting onto” methodology with H-PDMS, a template or backbone must be synthesized as depicted in Fig. 1 involving two distinct stages: a PS-*b*-PMVS diblock copolymer is prepared and coupled to a second living PS block. PS-*b*-PMVS formation occurs *via* anionic polymerization of sequential monomer addition starting with styrene in cyclohexane initiated by *sec*-BuLi. The initial styrene concentration is set to 3 w/v% to ensure diblock solubility based on preliminary experiments, as shown in Fig. S8.† After complete polymerization of styrene, a second monomer, D_3_V, that allows for grafting is added to the living PS to create the diblock copolymer. However, direct addition of D_3_V to the living PS leads to a high *Đ* = 1.56. Therefore, it is necessary to tune the crossover reaction to ensure narrow molecular weight distribution. SEC chromatograms of the PS block and its corresponding PS-*b*-PMVS diblock are shown in Fig. 3, where panels differ on the condition of crossover from PS to PMVS blocks. Polymerization conditions for the PS block are the same for all cases which leads to a narrow molecular weight distribution of *Đ* ≈ 1.08. However, the molecular weight distribution of PS-*b*-PMVS diblock copolymer is noticeably and adversely influenced by the reaction conditions when D_3_V is added, *i.e.* the solvent polarity and the nature of active ion. This dependency is clearly seen in Fig. 3a and b when THF is added after an overnight or just before the addition of D_3_V monomer, respectively. As opposed to D_3_, D_3_V is highly reactive such that polymerization can occur even without the presence of any promoter, THF, but many of the end ions are still in a dormant state with a slow exchange rate between dormant and active state, resulting in high dispersity. Upon addition of THF, chains grow rapidly at the same time, but from polydisperse oligomers which leads to a final multi-modal molecular weight distribution, as seen in Fig. 3a. When THF is added just before the D_3_V, the final molecular weight distribution becomes bimodal; a distribution corresponding to the initial homo polystyrene and another distribution from the resulting PS-*b*-PMVS diblock, as seen in Fig. 3b. This originates from the higher reaction rate of D_3_V monomers with silanolate ions rather than poly(styryl)lithium ions, which results in only a fraction of living polystyrenes propagating and a higher degree of polymerization for the PMVS block rather than the target molecular weight. To improve the fidelity of this crossover reaction, four units of D_3_ relative to the initiator are added to alter the nature of all active ions to silanolate ions to ensure similar reactivity of the propagating ions and the elimination of poly(styryl) ions. As D_3_ does not propagate in nonpolar hydrocarbon solvents but only transfers end ions of poly(styryl)lithium to silanolate ions,^36^ thus, D_3_ is allowed to react with living PS chains overnight followed by the addition of THF (10 v%) and D_3_V. However, a small portion of homo-PS still exists in its unreacted state and a shoulder peak is observed in SEC curve, as seen in Fig. 3c. To further improve the crossover reaction, a small amount of THF (1 v%) is added together with D_3_ to shift the ion association equilibrium towards active state, hence to increase the probability of reaction. Consequently, when D_3_V and more THF (10 v%) is added, all the active ions react with D_3_V in equal probability, resulting in a monomodal molecular weight distribution of the desired diblock as seen in Fig. 3d.

**Fig. 4:**
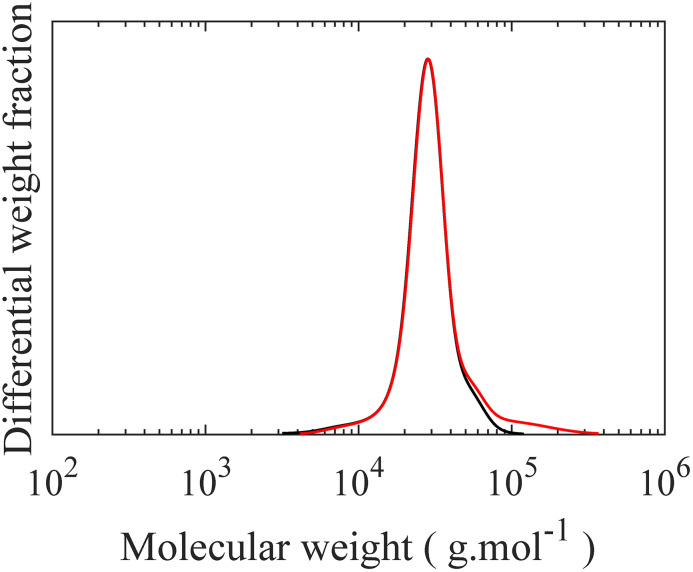
SEC curves of PS-*b*-PMVS diblock copolymer (**—**) before halogen exchange reaction containing benzyl chloride (BnCl) group, *M*_n_ = 25 400, *M*_w_ = 29 700 g mol^−1^, *Đ* = 1.17 and (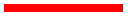) after halogen exchange reaction containing benzyl bromide (BnBr) group, *M*_n_ = 27 000, *M*_w_ = 35 300 g mol^−1^, *Đ* = 1.30.

However, a third PS block cannot be coupled directly to this PS-*b*-PMVS diblock by the same method of sequential monomer addition as a result of mismatch between the nucleophilicity of the growing anions and the electron affinity of the styrene monomer.^37^ Therefore, a targeted coupling reaction is needed between PS-*b*-PMVS diblock and another PS block using a heterobifunctional linking agent. The monomodal diblock is quenched with the linking agent, CMPDMS, to introduce a specific functional group at the end of obtained PS-*b*-PMVS diblock for further coupling to a second living PS block. CMPDMS selectively reacts with living PS-*b*-PMVS diblock due to the greater reactivity of the –Si(Me)_2_–Cl than of the –Ph–CH_2_–Cl group toward silanolate nucleophiles, leaving a functional benzyl chloride (BnCl) group at the end of diblock copolymer.^34,38^ To improve the probability of coupling between PS-*b*-PMVS diblock and a third block of PS, the resulting BnCl functional group is then transformed to the better leaving group, benzyl bromide (BnBr), by treating with LiBr in acetone/heptane (3/1).^39^ The ^1^H NMR spectrum shows a clear shift to 4.47 ppm, assigned to –Ph–CH_2_–Br, from the disappearance of the –Ph–CH_2_–Cl signal at 4.56, see Fig. S9;† end functionalization is calculated to be 76% based on the ratio of –Ph–CH_2_–Cl (4.56 ppm) to CH_3_ (0.9 ppm) signals. Additionally, SEC peaks of both polymers are found to be nearly identical before and after the halogen exchange reaction as shown in Fig. 4, confirming the conversion of BnCl to BnBr without any undesirable side reactions.

**Fig. 5:**
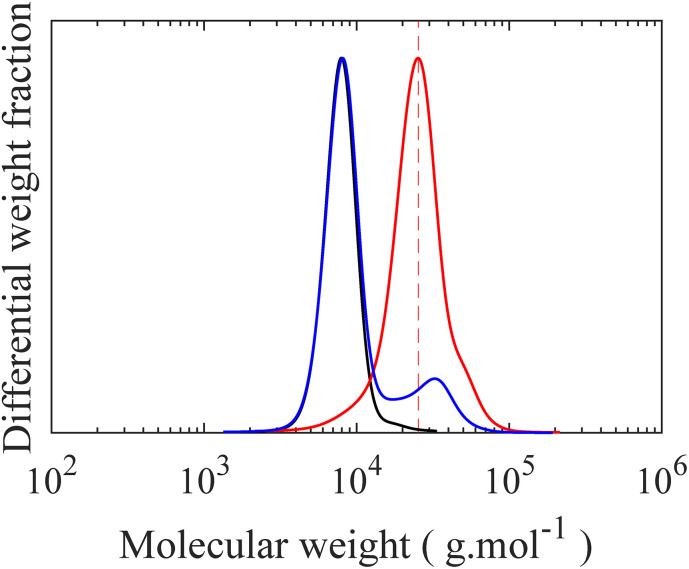
SEC curves of coupling reaction. (**—**) DPE-end functionalized PS, *M*_n_ = 7600, *M*_w_ = 8200 g mol^−1^, *Đ* = 1.08, (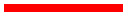) BnBr-functionalized diblock, *M*_n_ = 21 500, *M*_w_ = 27 100 g mol^−1^, *Đ* = 1.25, (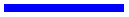) resulting triblock with excess PS.

To form the desired triblock from this Br-functionalized diblock copolymer, a DPE-end functionalized PS is prepared.^40^ To enhance the probability of the coupling, a five-fold molar excess of DPE-capped PS relative to BnBr-diblock is used. The SEC chromatograms after this coupling reaction displayed two distinct peaks. The position of a lower molecular weight peak matches precisely with the PS peak at 7900 g mol^−1^, corresponding to the excess PS used in the reaction. The diblock peak shifts from a molecular weight of 25 400 to 32 800 g mol^−1^, corresponding to the formation of the desired triblock by the coupling reaction as seen in Fig. 5. Crucially, this increase also demonstrates that no unwanted side reactions occur between the pendant vinyl groups of the middle block in the triblock and the excess PS during the coupling which would have resulted in higher molecular weights given the excess molarity. Additionally, detrimental chain cleavage in the triblock copolymer could also occur, thus end capping of living PS with DPE is a necessary step to reduce its reactivity which otherwise leads to chains scission during the coupling step; this undesirable chain scission is observed as a disappearance of the diblock peak in SEC curves, as seen in Fig. S10.† These crucial modifications to the synthesis of the backbone triblock prevent unwanted side reactions while permitting a high degree of monodispersity in the linear triblock and maintaining the vinyl functional groups in the middle block required for LBBL formation *via* “grafting onto”.

### 3.3 Grafting reaction

**Fig. 6:**
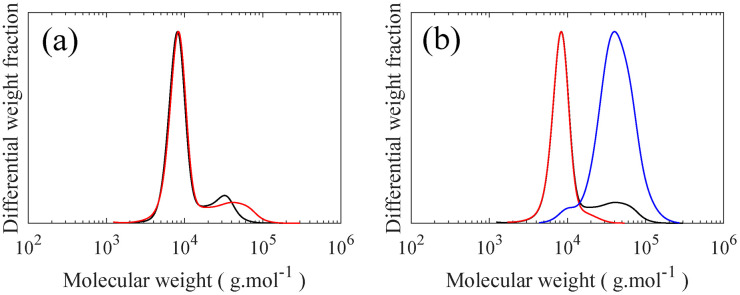
SEC curves for grafting and purification steps. (a) Grafting of H-PDMS on linear PS-*b*-PMVS-*b*-PS triblock copolymer. (**—**) Linear triblock copolymer with excess homo PS, (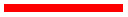) LBBL triblock copolymer in grafted state with excess homo PS and unreacted PDMS chains, (b) isolating desired LBBL triblock copolymer from excess homo PS and unreacted PDMS chains (**—**) product of grafting step (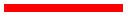) supernatant in precipitation step (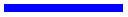) precipitant of precipitation step.

In the final step, grafting of H-PDMS side chains onto the PMVS middle block of the linear triblock through hydrosilylation reaction leads to PS-*b*-bbPDMS-*b*-PS polymers. This “grafting onto” approach features the possibility of making multiple topologies by varying the grafting density and side chain length, with the same triblock backbone, which significantly enhances consistency in materials properties. In this step, H-PDMS chains along with the product of the coupling reaction, which is a mixture of the desired PS-*b*-PMVS-*b*-PS triblock, PS-*b*-PMVS diblock, and excess homo PS, see Fig. S11,† are dissolved in anhydrous toluene and undergo a specific hydrosilylation reaction between silicone hydride and unsaturated vinyl groups in the presence of a platinum (Karstedt's) catalyst. During this reaction, many H-PDMS, each carrying one terminal hydride group, are grafted to the backbone acting as side chains. The grafting density of bottlebrush block can be tuned by changing the ratio of H-PDMS to linear backbone and monitored from ^1^H NMR spectra based on the ratio of consumed vinyl groups to the end CH_3_ groups of PDMS chains which do not contribute in the grafting reaction, see Fig. S12.† The SEC curve of linear triblock shifted to higher molecular weights after grafting, while the PS peak remains unchanged, confirming the grafting reaction, as seen in Fig. 6a. The resulting bottlebrushes are easily purified by precipitation as the molecular weight of bottlebrush chains, hence their solubility, differ significantly from the ungrafted, relatively low molecular weight H-PDMS and excess PS chains. Fig. 6b shows the SEC curves of starting material before precipitation along with both precipitant and supernatant after the precipitation step, showing that bottlebrush molecules are only present in the precipitant, proving a straightforward purification of desired bottlebrush chains. In this study, only one symmetric PS-*b*-bbPDMS-*b*-PS is synthesized, with the symmetric DP of PS blocks and PDMS middle block are 60 and 172, respectively. The grafted PDMS side chains have DP of 30 and the grafting density of bottlebrush block is 25%, meaning that there are on average 43 side chains on each bottlebrush molecule. These structural parameters are chosen to fabricate a bottlebrush physical network with elasticity on the order of biological tissues, however bottlebrushes with longer size chains and also higher grafting densities can be prepared with the same approach.

**Fig. 7:**
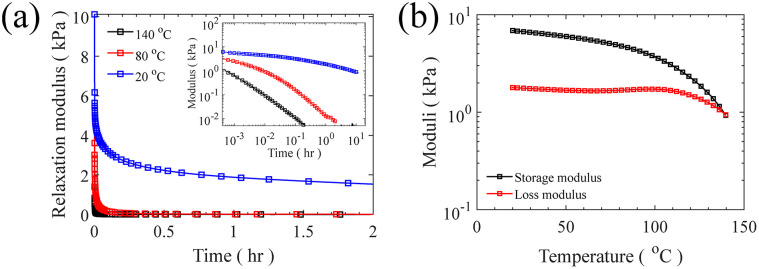
Mechanical response of bottlebrush sample (mixture of PS-*b*-bbPDMS and PS-*b*-bbPDMS-*b*-PS). (a) Shear relaxation modulus after a step strain of 5% at different temperatures of (**—**) 140 °C, (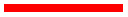) 80 °C, (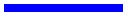) 20 °C (inset: log–log plot of relaxation modulus). (b) (**—**) Storage and (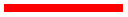) loss modulus in an oscillatory temperature sweep measurement at 1 Hz with 5% strain.

After isolating bottlebrush polymers, the ratio between bottlebrush diblock and triblock is quantified by comparing ^1^H NMR spectra of linear diblock copolymer and isolated bottlebrush chains. Knowing the molecular weight of both PS end blocks in triblock copolymer allows for a clear calculation of the fraction of triblock copolymer in the final material. For example, in this current case of symmetric PS end blocks, the ratio of styrenic protons to total initial vinyl protons doubles if all diblock chains become triblock. It should be noted that the total initial vinyl groups in bottlebrush chains can be recalculated considering the grafting density. In this study, the composition of final material is quantified as 28/72 triblock/diblock shown in Fig. S13;† This ratio directly affects the mesoscopic structure of this LBBL material being more physically crosslinked as the ratio of triblock increases. At the low weight fraction of PS prepared here, approximately 6.5%, LBBL copolymers are anticipated to self-assemble to a disordered spherical phase, regardless of the bottlebrush stiffness.^24^ The strength and relaxation of this elastic network is assessed using stress relaxation shear rheology test at different temperatures both higher and lower than the *T*_g_ of PS. A thermal glass transition of 73 °C is found for the linear PS-*b*-PMVS diblock while no clear transition is found for the final bottlebrush mixture due to the low mass fraction of PS present in this material, depicted in Fig. S16.† At 20 °C, the sample exhibits a stress relaxation modulus of 0.9 kPa while this reduces to 0 Pa at 80 °C and 140 °C, showing a solid to liquid transition by approaching the *T*_g_ of hard PS domains, as shown in Fig. 7a. The relaxation of the network at 20 °C, which is lower than the *T*_g_ of PS, is likely dominated by the presence of bottlebrush diblock copolymers and their spatial reorganization within the network to relax the imposed stress. Additionally, at 20 °C, a low strain, 5%, frequency sweep exhibits a near constant elastic modulus for low frequencies emphasizing the static mesoscopic network, shown in Fig. S15.† The absolute value of the constant network modulus is influenced by the ratio of the triblock copolymers in the material as main source of the elasticity in this physical network. The solid to liquid transition can also be seen in the oscillatory temperature sweep in Fig. 7b where loss modulus becomes higher than storage modulus around 140 °C in the test conditions. The low values of elastic moduli, on the order of 10 kPa, for the prepared LBBL copolymer are in a good agreement with reported values for similar materials in the literature.^24^

**Fig. 8:**
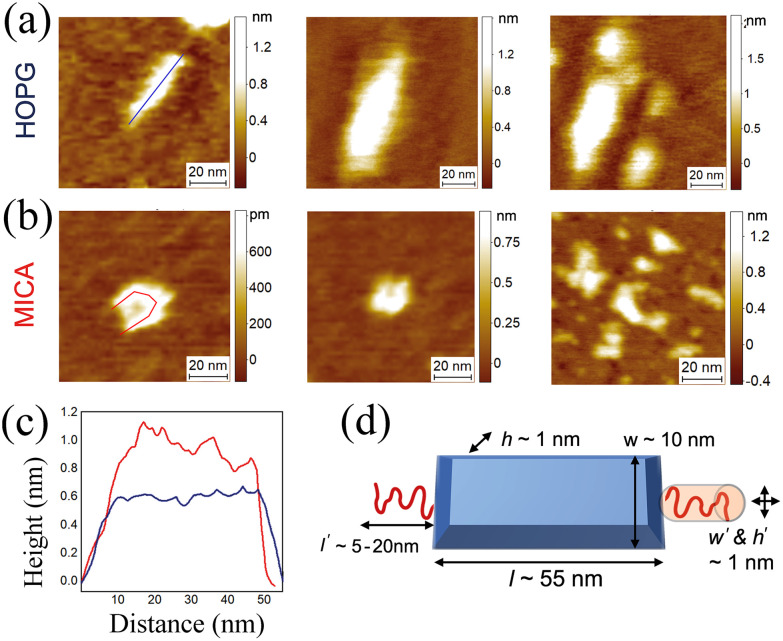
High-resolution AFM maps of bottlebrush sample (mixture of PS-*b*-bbPDMS and PS-*b*-bbPDMS-*b*-PS) on (a) highly ordered pyrolytic graphite (HOPG) and (b) mica. (c) Cross-sectional dimensions of some typical polymers on the surface with *ca.* 50–60 nm length and 0.5–1 nm height, the measured sizes well correspond with (d) the expected experimental design.

Bottlebrushes are macromolecules large enough to be experimentally visualized and their morphology and dimensions validated at the single molecule level by high-resolution and phase-controlled AFM height maps. We use two independent surfaces, atomically flat highly ordered pyrolytic graphite (HOPG) and mica substrates, as shown in Fig. 8. To avoid large imaging force and keep consistency in measured height between independent samples, a constant regime of phase change of tip oscillation is maintained within Δ20° during all AFM measurements.^29^ The bottlebrush middle block in the LBBL copolymer is clearly observed in these images with a contour length of ≈50–60 nm and a height of ≈0.5–1 nm. These results are in good agreement with the expected dimension based on the measured height of an organic molecule (several Angstroms), the degree of polymerization and known fact for a PDMS monomer with length of 0.3 nm.^10^ Isolated chains exhibit different surface conformations depending on the surface interaction with different substrate as shown on the left and the middle images in Fig. 8a and b; chains are more spread on hydrophobic HOPG substrate, resulting in a lower and uniform height profile accompanied with an elongated column-shaped conformation; while on negatively charged mica substrate, they are more in a folded conformation with higher and varied height profile in Fig. 8c. These observations can be ascribed to the strong attractive hydrophobic interaction of PDMS chains with the HOPG substrate, in contrast to the repulsive electrostatic interaction of non-charged PDMS chains with negatively-charged mica. Additionally, the different Hamaker constant of interaction between the tip and each different substrate in both experimental conditions results in a different underestimation of the real height of the polymer of ≈0.5–1 nm vertically, as typically observed for DNA.^30,31^ Then, also a lateral broadening of the shape of the polymer of ≈5–10 nm is expected due to the convolution effect of the AFM tip. However, feature dimensions are still clearly resolvable. Lastly in Fig. 8 where more than one chain exist, multiple deposited shapes or conformations are found which can be a result of self-assembly, as expected for these synthesized ABA triblock copolymers.

## 4 Conclusions

We have developed a new alternative method to fabricate PS-*b*-bbPDMS-*b*-PS triblock copolymers using a “grafting onto” approach where a high level of precision on the molecular structure is achieved by leveraging the separate anionic polymerization of side chains and backbone combined with a selective grafting reaction confirmed by high-resolution AFM. As oppose to the “grafting through” approach for synthesis of PDMS-based bottlebrushes, this proposed method results in a PDMS bottlebrush block with identical chemical composition for side chains and backbone, providing the possibility to further study the effects of backbone nature on network mechanical properties. Apart from versatility of synthesis, employing a “grafting onto” approach can allow for multiple bottlebrush topologies with an identical backbone to minimize batch-to-batch differences. This consequently improves the consistency in mechanical properties. Additionally, starting from a common backbone is a benefit from a practical point of view when seeking a scalable process to fabricate grams to kilograms of material. Another important novel feature of this synthesis method is the ability to accurately quantify the composition of bottlebrush chains, *i.e.* the ratio of diblock (LBB) and triblock bottlebrush (LBBL), as the presence of diblock bottlebrush plasticizes the final material by diluting the elastic network. Moreover, this approach lacks permanent crosslinks between bottlebrushes as the elasticity emanates from the microphase separated high *T*_g_ PS domains that could dissociate at high temperature. Thus these soft LBBL elastomers experience a temperature-triggered solid-to-liquid transition which makes them functional candidates for 3D printing of soft and strain-stiffening tissue scaffolds, which is currently being explored for future application.

## Conflicts of interest

There are no conflicts to declare.

## Supplementary Material

PY-013-D2PY00841F-s001

PY-013-D2PY00841F-s002

PY-013-D2PY00841F-s003

PY-013-D2PY00841F-s004

PY-013-D2PY00841F-s005

PY-013-D2PY00841F-s006

PY-013-D2PY00841F-s007

PY-013-D2PY00841F-s008

PY-013-D2PY00841F-s009

PY-013-D2PY00841F-s010

PY-013-D2PY00841F-s011

PY-013-D2PY00841F-s012

PY-013-D2PY00841F-s013

PY-013-D2PY00841F-s014

PY-013-D2PY00841F-s015

PY-013-D2PY00841F-s016

PY-013-D2PY00841F-s017

PY-013-D2PY00841F-s018
