# Supplementary material for: Synthesis of well-defined linear–bottlebrush–linear triblock copolymer towards architecturally-tunable soft materials
Source: Polym Chem. 2022 Jul 20;13(32):4666–74. doi: 10.1039/d2py00841f (PMC9379773; doi:10.1039/d2py00841f)
Supplement: PY-013-D2PY00841F-s001 [file PY-013-D2PY00841F-s001.pdf]

Electronic Supplementary Material (ESI) for Polymer Chemistry.
This journal is © The Royal Society of Chemistry 2022

Supporting Information: Synthesis of Well-defined Linear-Bottlebrush-Linear Triblock Copolymer Towards Architecturally-tunable Soft Materials

Vahid Asadi,[†] Xuecong Li,[†,‡] Francesco Simone Ruggeri,[†,‡] Han Zuilhof,[‡,¶] Jasper van der Gucht,[†] and Thomas E. Kodger*[,†]

†*Physical Chemistry and Soft Matter, Wageningen University & Research, Stippeneng 4, Wageningen 6708 WE, The Netherlands*

‡*Laboratory of Organic Chemistry, Wageningen University & Research, Stippeneng 4, Wageningen 6708 WE, The Netherlands*

¶*Department of Chemical and Materials Engineering, Faculty of Engineering, King Abdulaziz University, 21589 Jeddah, Saudi Arabia*

E-mail: thomas.kodger@wur.nl

Part 1: Information on synthesis of H-PDMS side chains

Fig. S1-S3: ^{1}H NMR spectra of H-PDMS side chains polymerized in presence of 10% THF until full monomer conversion.

Fig. S4: SEC chromatograms of H-PDMS side chains with various lengths proceeded to full monomer conversion.

Fig. S5: Kinetic study for polymerization of H-PDMS proceeded to 50% monomer conversion.

Fig. S6-S7: ^{1}H NMR spectra of H-PDMS side chains polymerized in presence of 10% THF until 50% monomer conversion.

Part 2: Information on synthesis of linear PS-*b*-PMVS-*b*-PS triblock copolymer

Fig. S8: SEC results of preliminary experiments on styrene polymerization in different conditions.

Fig. S9: ^{1}H NMR spectra for halogen exchange reaction.

Fig. S10: SEC results of failed approaches for the coupling of PS block to PS-*b*-PMVS diblock.

Fig. S11: ^{1}H NMR spectrum for the product of the coupling reaction.

Part 3: Information on synthesis of LBBL PS-*b*-bbPDMS-*b*-PS triblock copolymer and its mechanical response

Fig. S12: ^{1}H NMR spectra before and after the grafting step to calculate grafting density.

Fig. S13: ^{1}H NMR spectra for linear diblock and isolated bottlebrush chains to determine the final composition of material.

Fig. S14-S15: Mechanical response of the synthesized bottlebrush sample.

Part 4: Thermal analysis

Fig. S16-S17: DSC and TGA curves of the PS-*b*-PMVS diblock and the synthesized bottlebrush sample.

Part 1: Information on synthesis of H-PDMS side chains

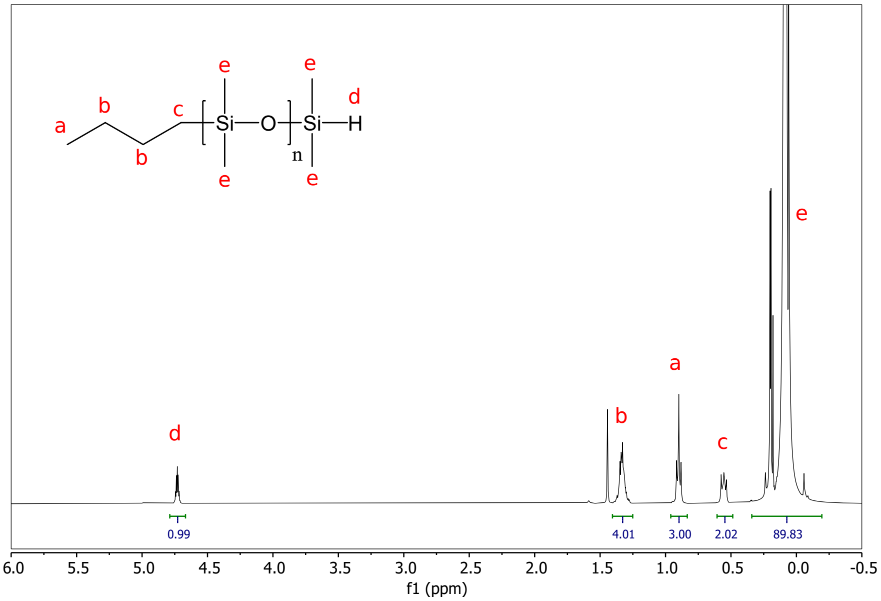

Fig. S1: ^{1}H NMR spectrum of monohydride-terminated polydimethylsiloxane (H-PDMS) with theoretical DP of 15 ; polymerized in presence of 10% THF until full conversion. ^{1}H NMR (400 MHz, $CDCl_3$, 25°C): δ = 0.08 *ppm* (m, 3 H; $-Si-CH_3$), 0.55 *ppm* (t, 2 H, $-CH_2-$), 0.89 *ppm* (t, 3 H, $-CH_3$), 1.33 *ppm* (m, 4 H, $-CH_2-CH_2-$), 4.73 *ppm* (m, 1 H, $-Si-H$). DP $= \frac{e}{a \times 2} = \frac{89.83}{3 \times 2} \approx 15$. $M_n = \frac{e}{a \times 6} \times$ molar mass of $D_3 = \frac{89.83}{3 \times 6} \times 222.46 \approx 1110$ $g.mol^{-1}$.

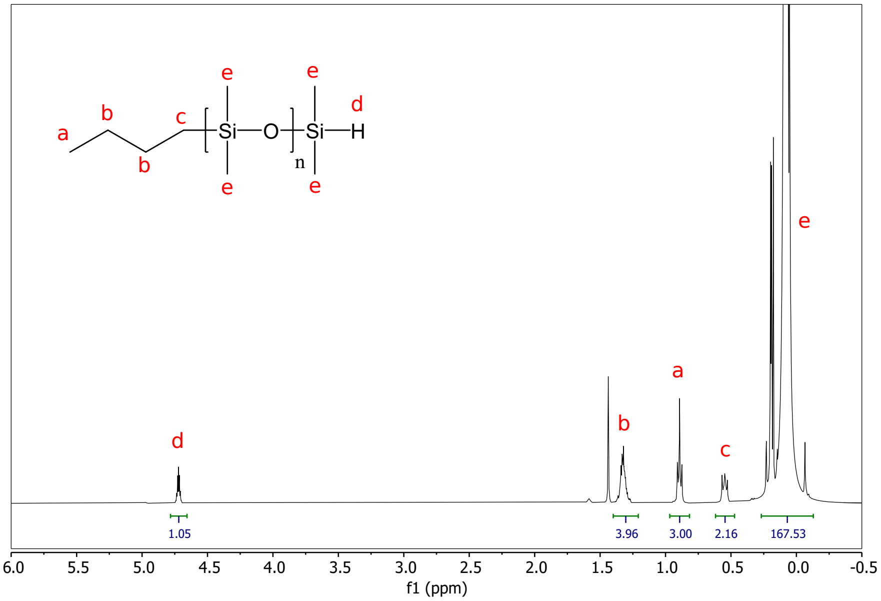

Fig. S2: ^{1}H NMR spectrum of monohydride-terminated polydimethylsiloxane (H-PDMS) with theoretical DP of 30 ; polymerized in presence of 10% THF until full comversion. ^{1}H NMR (400 MHz, $CDCl_3$, 25°C): δ = 0.08 *ppm* (m, 3 H; $-Si-CH_3$), 0.55 *ppm* (t, 2 H, $-CH_2-$), 0.89 *ppm* (t, 3 H, $-CH_3$), 1.32 *ppm* (m, 4 H, $-CH_2-CH_2-$), 4.72 *ppm* (m, 1 H, $-Si-H$). DP $= \frac{e}{a\times 2} = \frac{167.53}{3\times 2} \approx 28$. $M_n = \frac{e}{a\times 6}\times$ molar mass of $D_3 = \frac{167.53}{3\times 6}\times 222.46 \approx 2070$ g.mol^{-1}.

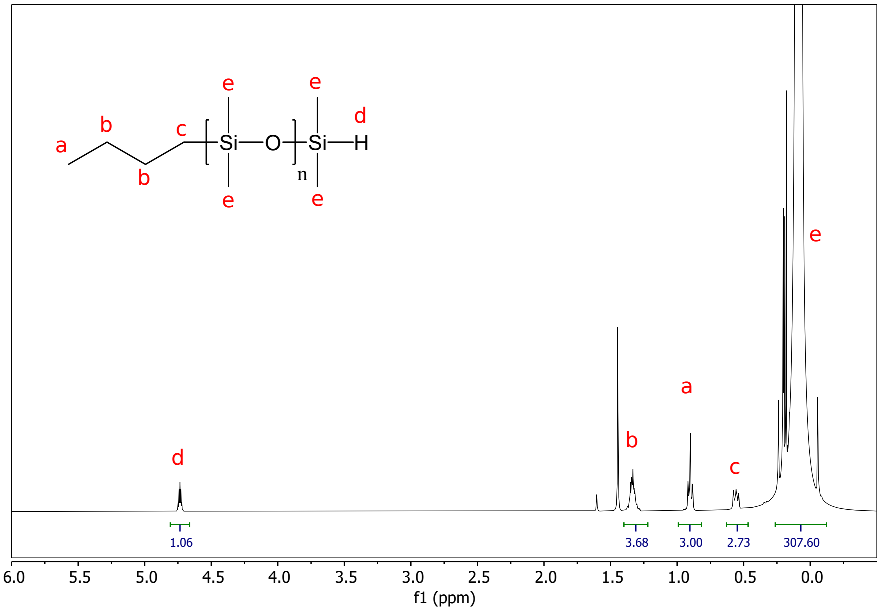

Fig. S3: ^{1}H NMR spectrum of monohydride-terminated polydimethylsiloxane (H-PDMS) with theoretical DP of 60 ; polymerized in presence of 10% THF until full comversion. ^{1}H NMR (400 MHz, $CDCl_3$, 25°C): δ = 0.09 *ppm* (m, 3 H; $-Si-CH_3$), 0.55 *ppm* (t, 2 H, $-CH_2-$), 0.89 *ppm* (t, 3 H, $-CH_3$), 1.33 *ppm* (m, 4 H, $-CH_2-CH_2-$), 4.73 *ppm* (m, 1 H, $-Si-H$). DP $= \frac{e}{a\times 2} = \frac{307.60}{3\times 2} \approx 51$. $M_n = \frac{e}{a\times 6}\times$ molar mass of $D_3 = \frac{307.60}{3\times 6}\times 222.46 \approx 3800$ $g.mol^{-1}$.

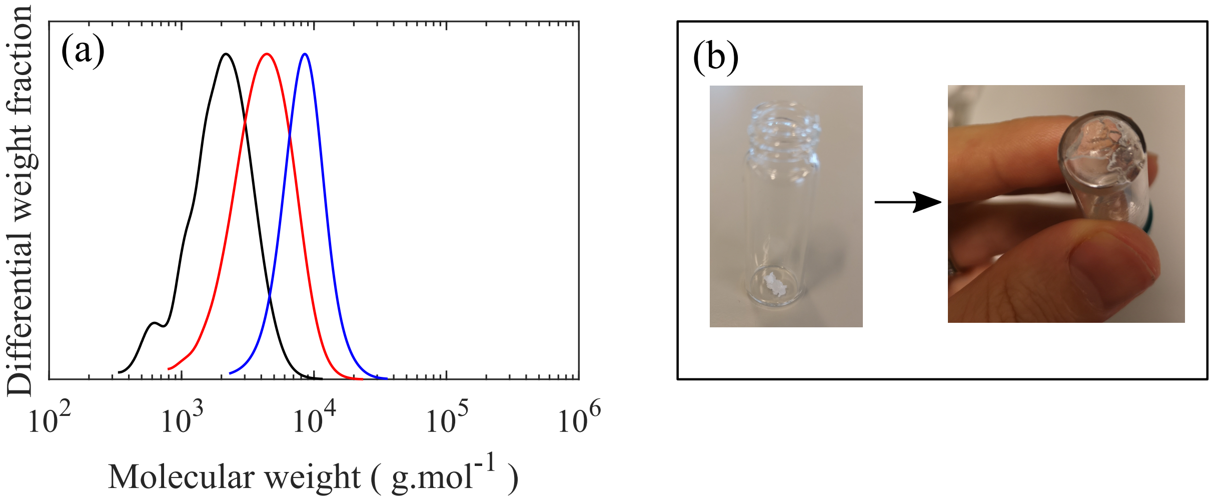

Fig. S4: **(a)** SEC chromatograms of H-PDMS with different molecular weights where polymerization proceeded to full conversion (—) M_n = 1630, M_w = 2220 $g.mol^{-1}$, Đ = 1.36, (—) M_n = 3520, M_w = 4620 $g.mol^{-1}$, Đ = 1.31, and (—) M_n = 7590, M_w = 8890, Đ = 1.17. **(b)** Using these H-PDMS chains for grafting reaction resulted in bottlebrush that swells in toluene due to presence of crosslinks.

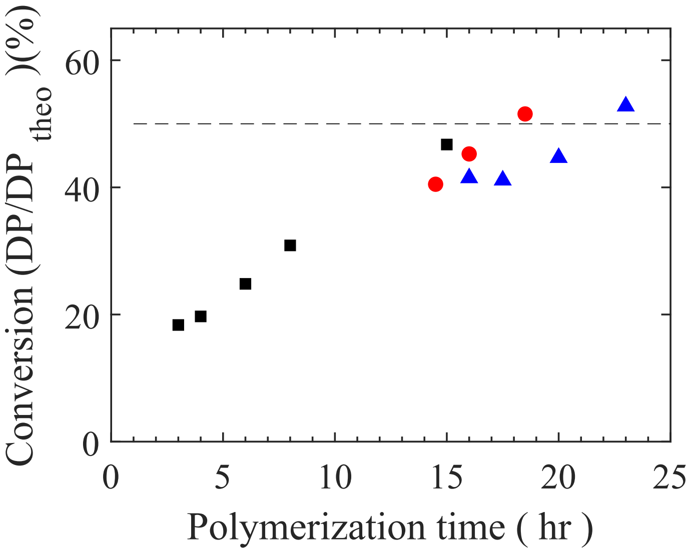

Fig. S5: Kinetic study for polymerization of H-PDMS. (■) DP_{theo} = 30 starting with 5 g monomer, (●) DP_{theo} = 30 starting with 20 g monomer, and (▲) DP_{theo} = 60 starting with 20 g monomer. All polymerizations were done in presence of 10% THF at 10 (w/v%) initial monomer concentration. Dash line corresponds to the aimed 50% conversion.

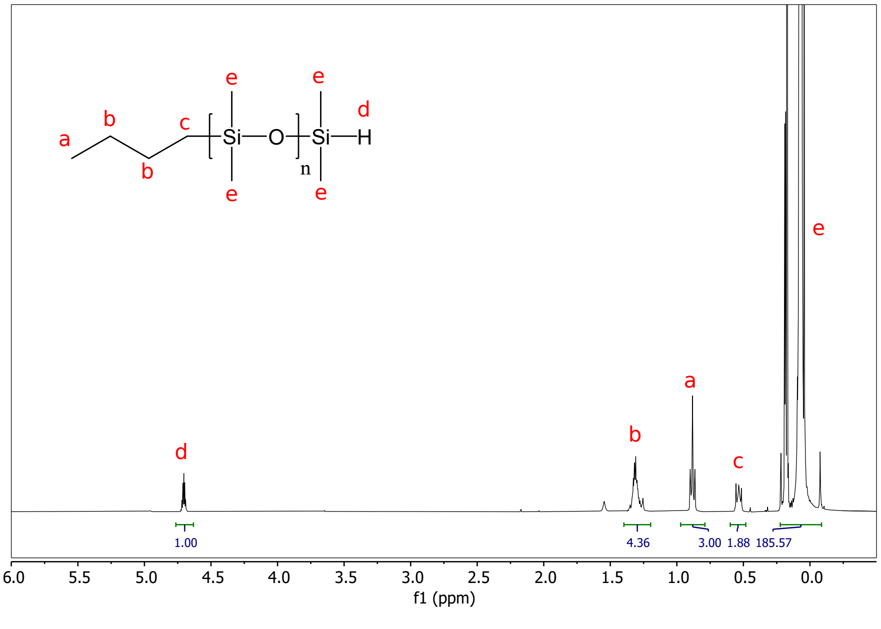

Fig. S6: ^{1}H NMR spectrum of monohydride-terminated polydimethylsiloxane (H-PDMS) with theoretical DP of 30 ; polymerized in presence of 10% THF until around 50% conversion. ^{1}H NMR (400 MHz, $CDCl_3$, 25°C): $\delta = 0.07$ *ppm* (m, 3 H; $-Si-CH_3$), 0.53 *ppm* (t, 2 H, $-CH_2-$), 0.88 *ppm* (t, 3 H, $-CH_3$), 1.30 *ppm* (m, 4 H, $-CH_2-CH_2-$), 4.70 *ppm* (m, 1 H, $-Si-H$). $DP = \frac{e}{a\times2} = \frac{185.57}{3\times2} \approx 30$. $M_n = \frac{e}{a\times6}\times$ molar mass of $D_3 = \frac{185.57}{3\times6}\times 222.46 \approx 2290$ $g.mol^{-1}$.

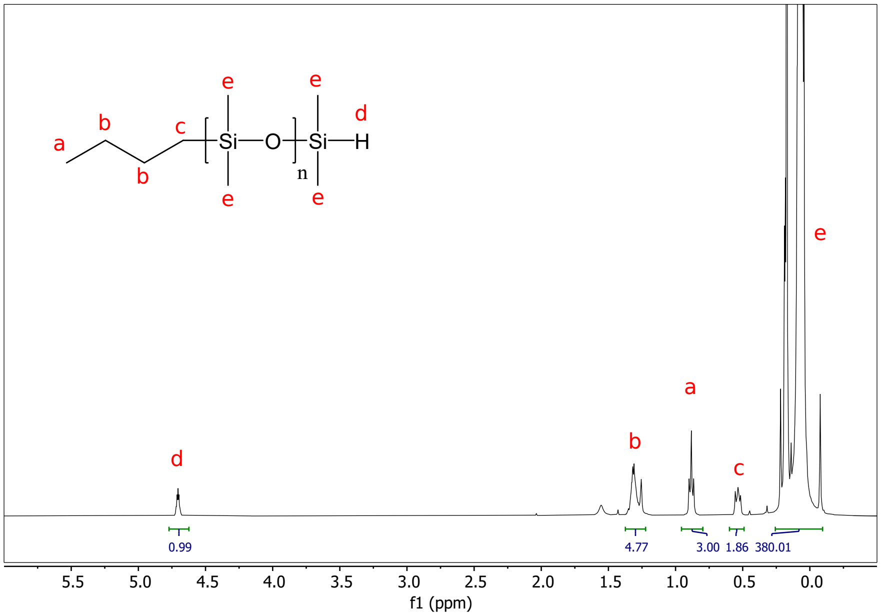

Fig. S7: ^{1}H NMR spectrum of monohydride-terminated polydimethylsiloxane (H-PDMS) with theoretical DP of 60 ; polymerized in presence of 10% THF until around 50% conversion. ^{1}H NMR (400 MHz, $CDCl_3$, 25°C): $\delta = 0.07$ *ppm* (m, 3 H; $-Si-CH_3$), 0.53 *ppm* (t, 2 H, $-CH_2-$), 0.88 *ppm* (t, 3 H, $-CH_3$), 1.30 *ppm* (m, 4 H, $-CH_2-CH_2-$), 4.70 *ppm* (m, 1 H, $-Si-H$). DP $= \frac{e}{a\times 2} = \frac{380.01}{3\times 2} \approx 63$. $M_n = \frac{e}{a\times 6}\times$ molar mass of $D_3 = \frac{380.01}{3\times 6}\times 222.46 \approx 4690$ g.mol^{-1}.

Part 2: Information on synthesis of linear PS-*b*-PMVS-*b*-PS triblock copolymer

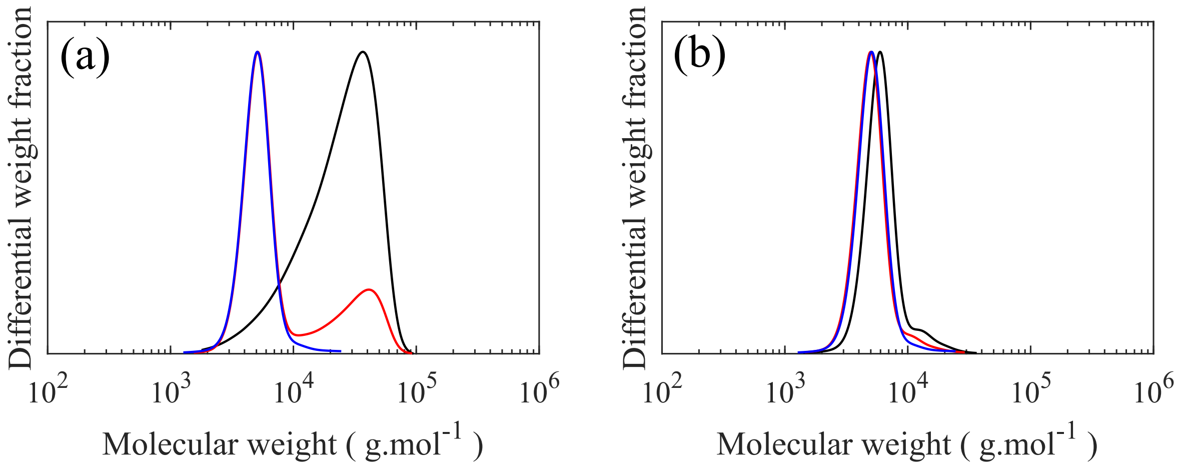

Fig. S8: SEC chromatograms of polystyrene. **(a)** Polymerizations were done with initial monomer concentration of 5 w/v% initiated by (—) n-BuLi in cyclohexane, M_n = 16900, M_w = 27800 g.mol^{-1}, Đ = 1.64 (—) n-BuLi in cyclohexane/THF 98/2, M_n = 6300, M_w = 13300 g.mol^{-1}, Đ = 2.11 and (—) sec-BuLi in cyclohexane, M_n = 4800, M_w = 5300 g.mol^{-1}, Đ = 1.10. **(b)** Polymerizations initiated by sec-BuLi with different initial monomer concentration (—) 3 w/v%, M_n = 5800, M_w = 6600 g.mol^{-1}, Đ = 1.14 (—) 4 w/v%, M_n = 4800, M_w = 5400 g.mol^{-1}, Đ = 1.12, and (—) 5 w/v%, M_n = 4800, M_w = 5300 g.mol^{-1}, Đ = 1.10. In all cases polymerizations stopped after 4 hours.

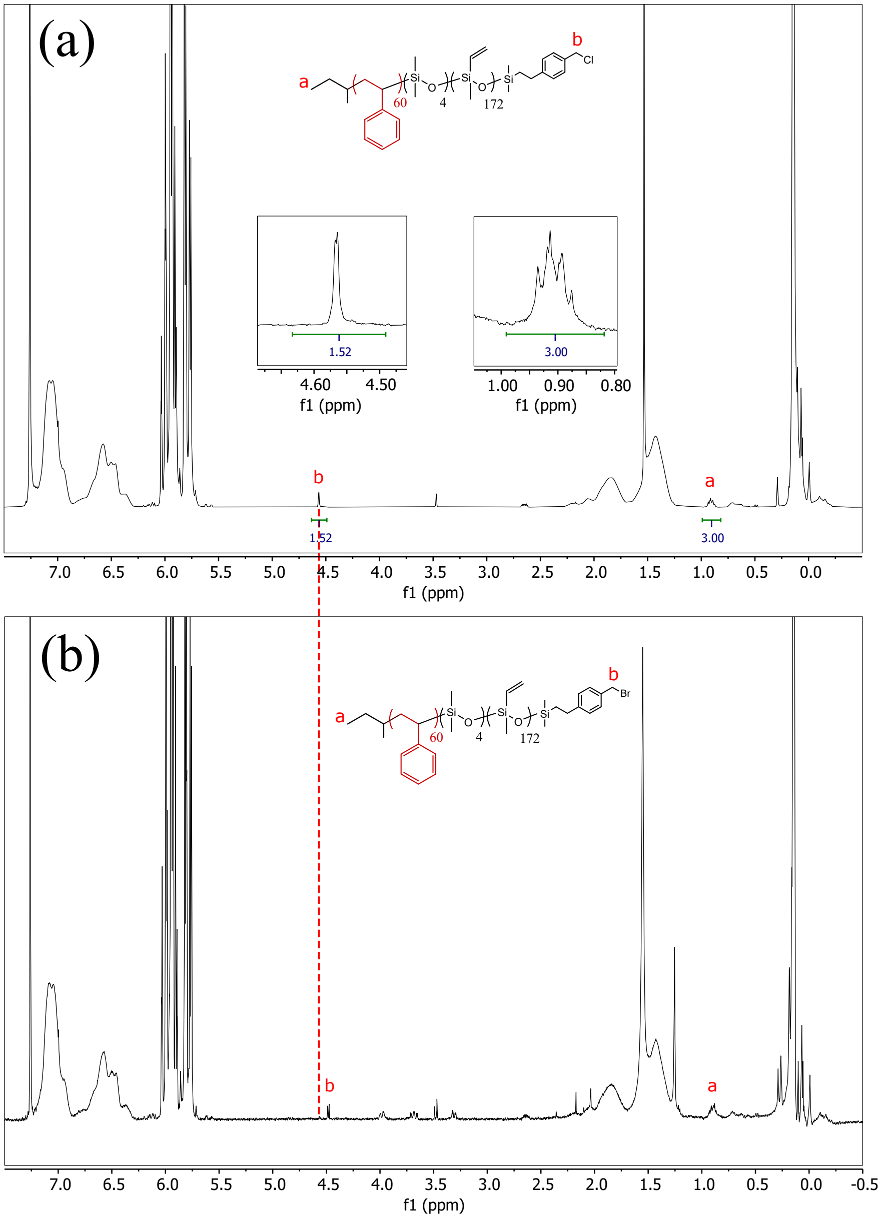

Fig. S9: ^{1}H NMR spectra for halogen exchange reaction. **(a)** BnCl-functionalized PS-*b*-PMVS diblock copolymer **(b)** BnBr-functionalized PS-*b*-PMVS diblock copolymer after halogen exchange. ^{1}H NMR (400 MHz, $CDCl_3$, 25°C).

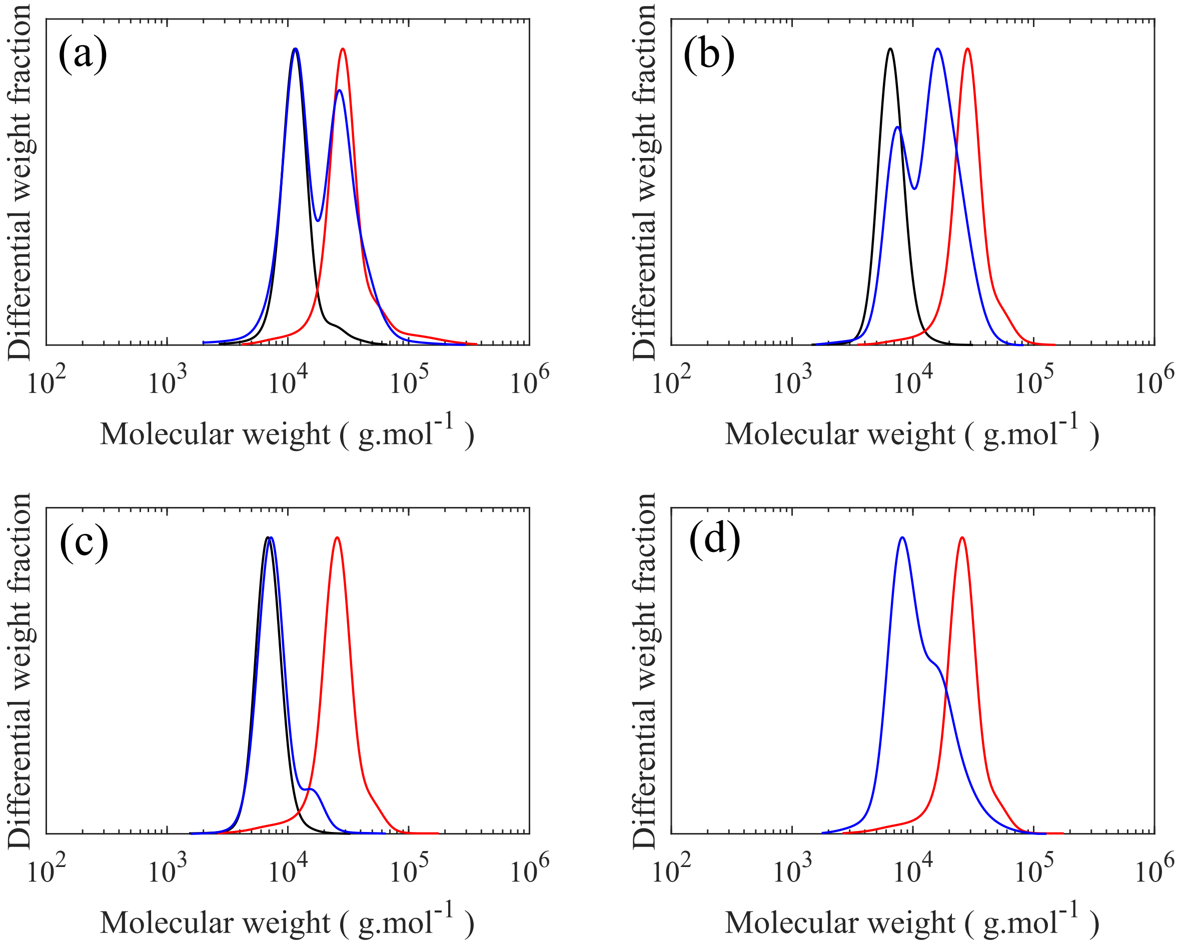

Fig. S10: SEC chromatograms of coupling reaction using various approaches. **(a)** and **(b)** coupling of (—) BnBr-functionalized PS-*b*-PMVS diblock copolymer with two different length of (—) living PS in THF/cyclohexane 50/50 (v/v) **(c)** coupling of (—) BnCl-functionalized PS-*b*-PMVS diblock copolymer with (—) living PS in presence of CsI in THF/DMF/cyclohexane 40/40/20 (v/v) **(d)** polymerization of styrene initiaed by (—) BnCl-functionalized PS-*b*-PMVS diblock copolymer through ATRP. Polymerization was carried out in solution of toluene/styrene 80/20 (v/v) with $[I]_0 : [M]_0 : CuCl : dNbpy = 1 : 570 : 2 : 4$ at 90°C for an overnight. In this approach, homo PS is formed as a result of styrene self initiation. In all plots (—) is the chromatogram for the product of coupling reaction. Because of presence of reactive living PS, it seems that chains scission occurs in diblock between PS and PMVS blocks as the diblock peak is not visible after the coupling reaction. However, a new peak at lower molecular weights than diblock appears which could be the PS blocks coupled together as its molecular weight is almost twice of the single PS block.

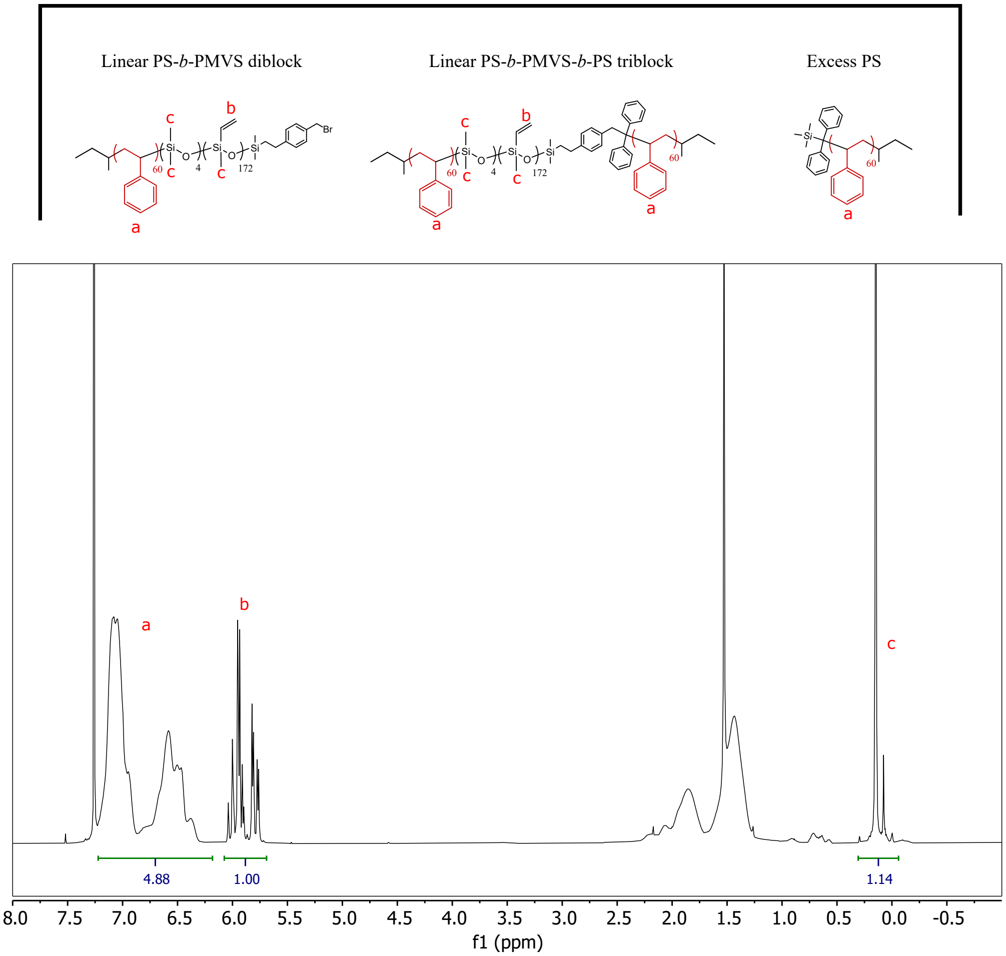

Fig. S11: ^{1}H NMR spectrum for the product of the coupling reaction which is a mixture of linear PS-*b*-PMVS diblock, PS-*b*-PMVS-*b*-PS triblock, and inert PS. ^{1}H NMR (400 MHz, $CDCl_3$, 25°C).

Part 3: Information on synthesis of LBBL PS-*b*-bbPDMS-*b*-PS triblock copolymer and its mechanical response

Calculation of the grafting density:

The PMVS block has a vinyl functional group on each repeating unit. During the grafting reaction, the monohydride-terminated PDMS chains attach to the PMVS block by hydrosilylation reaction between the end hydride group of the H-PDMS chains and vinyl functional group of the PMVS block in presence of Karstedt's catalyst. Therefore, as grafting reac-

tion continues, the amount of vinyl groups decreases. The reduction percentage of the vinyl groups provide us the grafting density. To calculate reduction percentage in the amount of vinyl groups, we take an aliquot from the reaction medium for ^{1}H NMR measurement before addition of the catalyst and use this sample as our reference point, shown in Fig. S12a. Then we add the catalyst and the grafting reaction begins, leading to consumption of vinyl groups. To quantify this reduction, we use the peak for the methyl CH_3 group located on the other end of H-PDMS chains, which originates from the n-BuLi initiator, as our reference peak, shown in the Fig. S12 with the letter "a" (0.88 ppm). We use this peak for the reference peak because it does not change during the grafting reaction and also it is a clear resolved peak that does not overlap with other peaks in the ^{1}H NMR spectrum.

$$\text{Grafting density (\%)} = \text{consumption of vinyl groups} = \frac{8.02-6.01}{8.02} \times 100 \approx 25\ (\%).$$

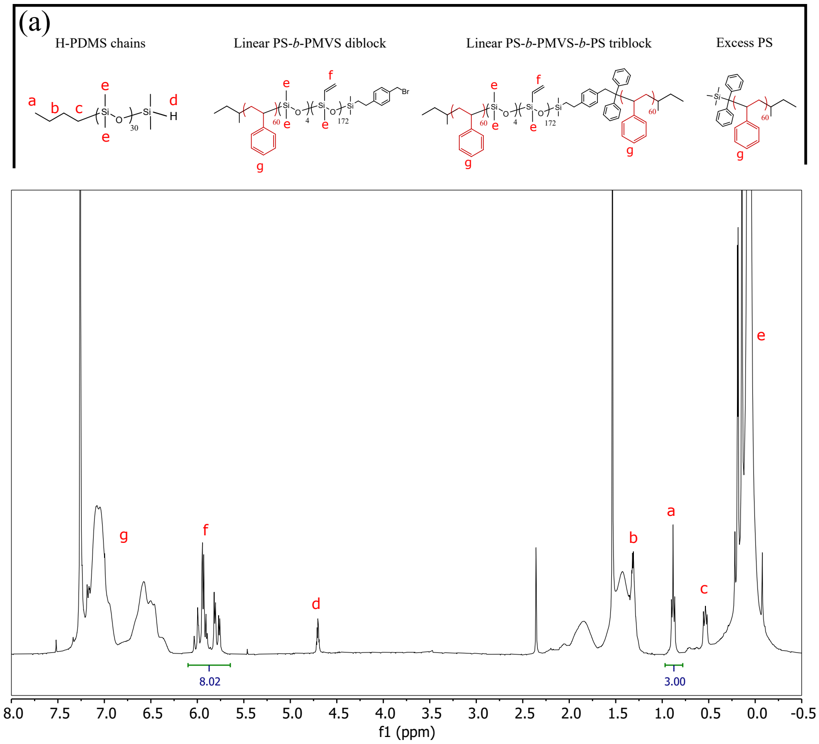

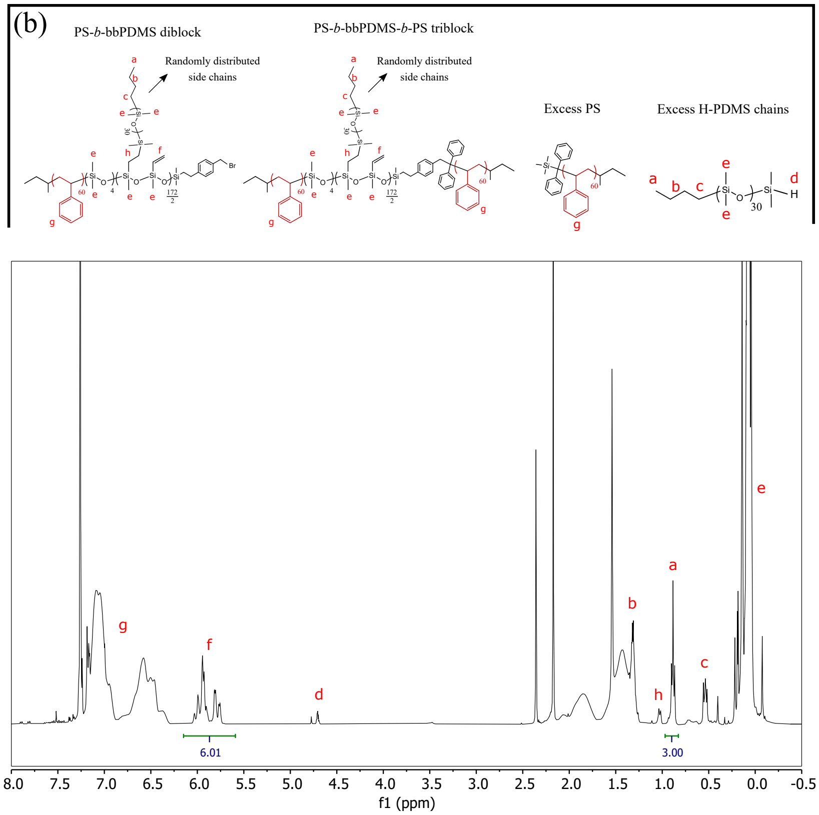

Fig. S12: ^{1}H NMR spectra for grafting reaction. **(a)** Initial point after mixing of H-PDMS chains with product of coupling step **(b)** after 14 days from start of grafting reaction. ^{1}H NMR (400 MHz, $CDCl_3$, 25°C). Grafting density (%) = consumption of vinyl groups = $\frac{8.02-6.01}{8.02} \times 100 \approx 25$ (%)

Calculation of the graft number:

To determine the average number of grafted side chains, DP of PMVS block needs to be determined. DP of PMVS block is determined based on ratio of the ring protons of styrene and vinyl protons ($H_{styrenic}/H_{vinyls} = 0.58$, shown in Fig. S13a), while DP of PS block is determined by the molecular weight values obtained from SEC for PS shown in Fig. 3d of the main manuscript.

$$DP_{PS} = \frac{M_n\ from\ SEC}{Molar\ mass\ of\ styrene} = \frac{6300}{104.15} \approx 60$$

$$DP_{PMVS} = DP_{PS} \times \frac{H_{vinyls}}{H_{styrenic}} \times \frac{number\ of\ H_{styrenic}\ in\ each\ unit\ of\ PS\ block}{number\ of\ H_{vinyls}\ in\ each\ unit\ of\ PMVS\ block}$$

$$DP_{PMVS} = 60 \times \frac{1}{0.58} \times \frac{5}{3} \approx 172$$

By knowing the grafting density and also the DP of PMVS block, the graft number is calculated as follow:

$$Graft\ number = grafting\ density \times DP_{PMVS} = 0.25 \times 172 \approx 43$$

Calculation of PS-*b*-bbPDMS-*b*-PS triblock fraction:

Fig. S13a shows the ^{1}H NMR spectrum for the linear PS-*b*-PMVS diblock copolymer. The broad peak located at 6.25-7.25 ppm corresponds to the five ring protons of styrene, labelled with letter "g" in the chemical structure. The peak located at 5.72-6.10 ppm corresponds to the three protons of vinyl group in each repeating unit of the PMVS block. In the PS-*b*-PMVS diblock copolymer, the ratio of styrenic ring protons to the protons in the vinyl

group is 0.58, shown in Fig. S13a. However, this ratio increases by linking of another PS block to the other end of the chain which is our logic to calculate the fraction of triblock copolymer in the final product. Fig. S13b shows the ^{1}H NMR spectrum for the mixture of diblock (PS-*b*-bbPDMS) and triblock (PS-*b*-bbPDMS-*b*-PS) in the bottlebrush state. Since 25% of the vinyl groups have been consumed during the grafting reaction, the vinyl peak in ^{1}H NMR spectrum (5.72-6.10 ppm) corresponds to the remaining vinyl groups which are 75% of the initial vinyl groups, used as the reference peak in Fig. S13b. Therefore, it is necessary to account for the grafting density before comparing with the linear PS-*b*-PMVS diblock.

$$Increase\ in\ the\ ratio\ of\ \frac{H_{\mathrm{styrenic}}}{H_{\mathrm{initial\ vinyls}}} = 0.743 - 0.58 = 0.163$$

This increase in the amount of styrenic ring protons originates from presence of triblock copolymer. By adding symmetric PS end block which is the case in this paper, if all the diblock chains had converted to the triblock, the ratio of $\mathrm{H_{styrenic}/H_{initial\ vinyls}}$ would have increased to 0.58. So, the fraction of triblock can be calculated as:

$$Fraction\ of\ triblock\ = \frac{increase\ in\ the\ ratio}{maximum\ possible\ increase} = \frac{0.163}{0.58} \approx 0.28$$

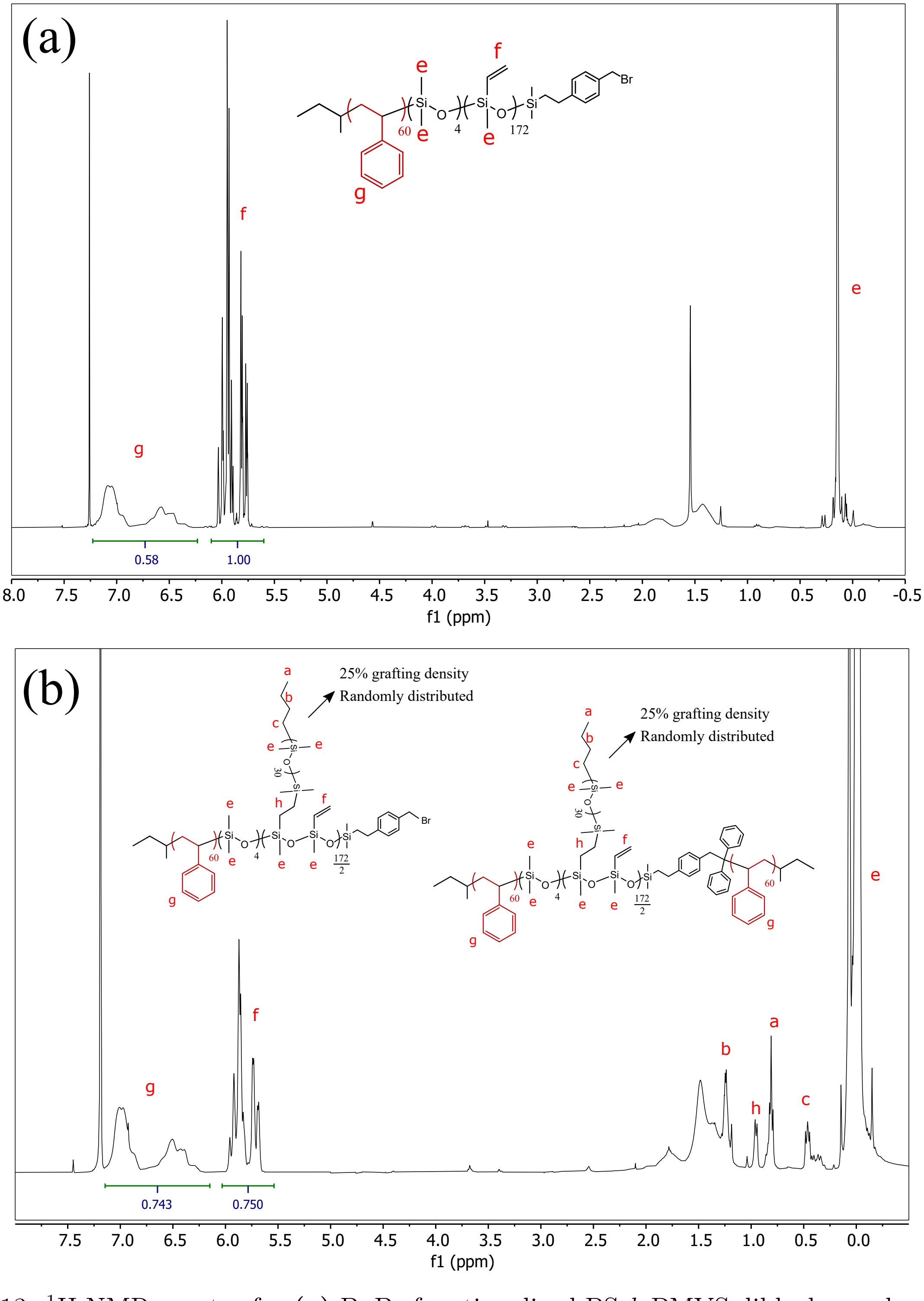

Fig. S13: ^{1}H NMR spectra for **(a)** BnBr-functionalized PS-*b*-PMVS diblock copolymer **(b)** purified bottlebrush diblock (PS-*b*-bbPDMS) and triblock (PS-*b*-bbPDMS-*b*-PS) copolymer. ^{1}H NMR (400 MHz, $CDCl_3$, 25°C).

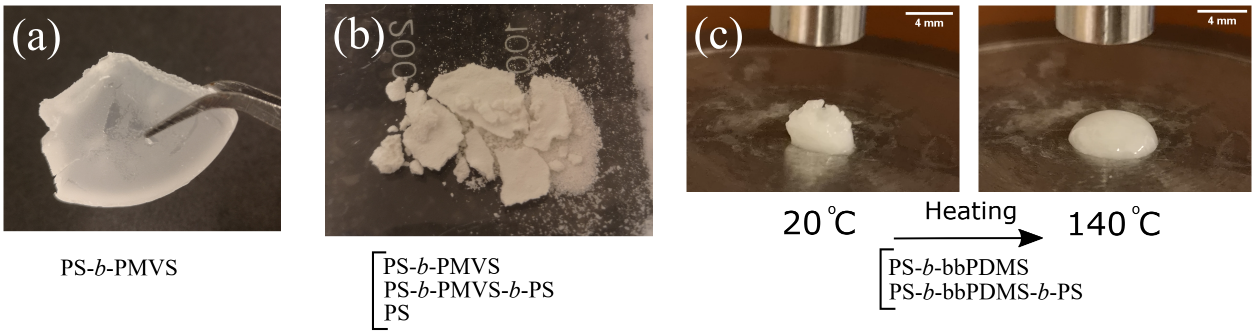

Fig. S14: Physical state of the synthesized materials in different steps. **(a)** Linear PS-*b*-PMVS diblock (not brittle, can be hold by tweezer with no deformation), **(b)** product of the coupling reaction including linear PS-*b*-PMVS diblock, PS-*b*-PMVS-*b*-PS triblock, and PS (brittle solid), **(c)** final product which is a mixture of bottlebrush diblock (PS-*b*-bbPDMS) and triblock (PS-*b*-bbPDMS-*b*-PS) copolymer (soft elastic material). Melting of the bottlebrush sample by heating in the course of loading on the rheometer, clearly showing the solid-to-liquid transition. The material is not completely transparent due to structural defects since it is a mixture of bottlebrush diblocks and triblocks which form clusters in the size range of wavelength of the visible light, leading to scattering events.

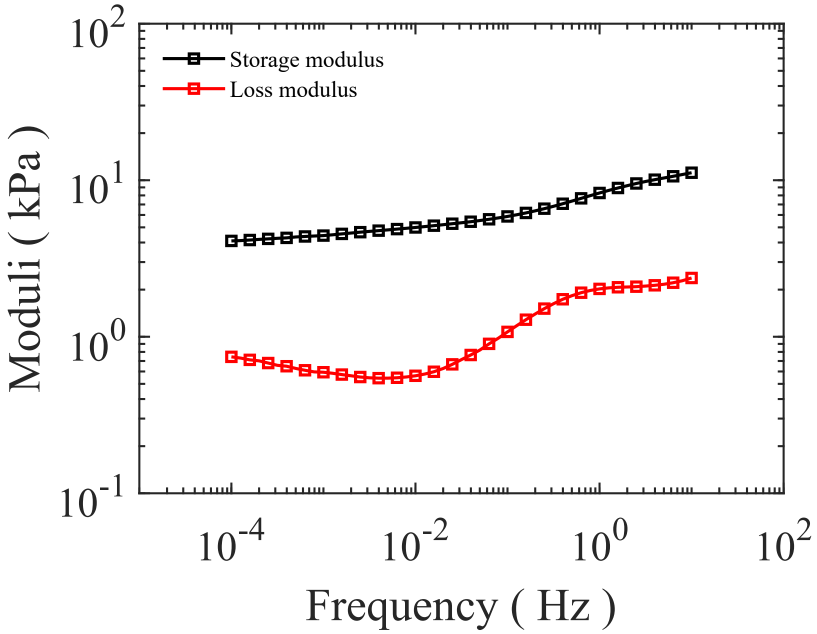

Fig. S15: Frequency sweep response of the mixture of bottlebrush diblock (PS-*b*-bbPDMS) and triblock (PS-*b*-bbPDMS-*b*-PS) copolymer at 20°C with 5 % strain, showing a constant storage modulus at low frequencies which proves the network formation in the sample.

Part 4: Thermal analysis

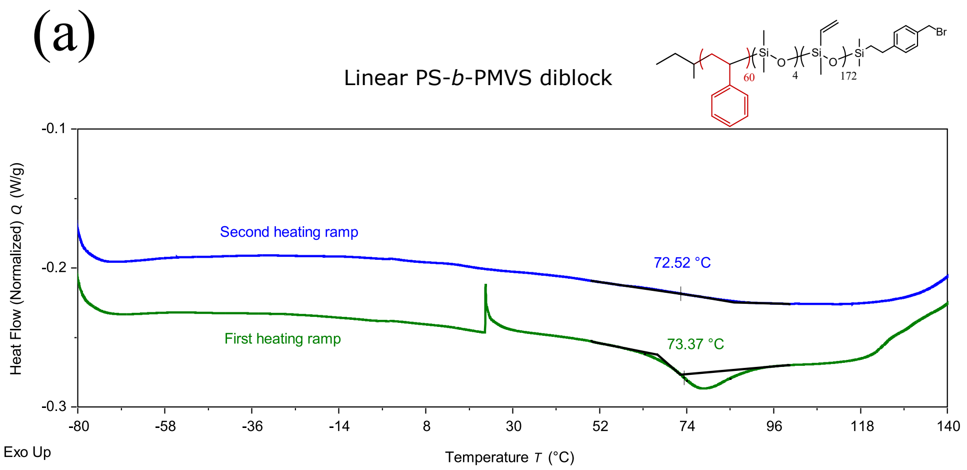

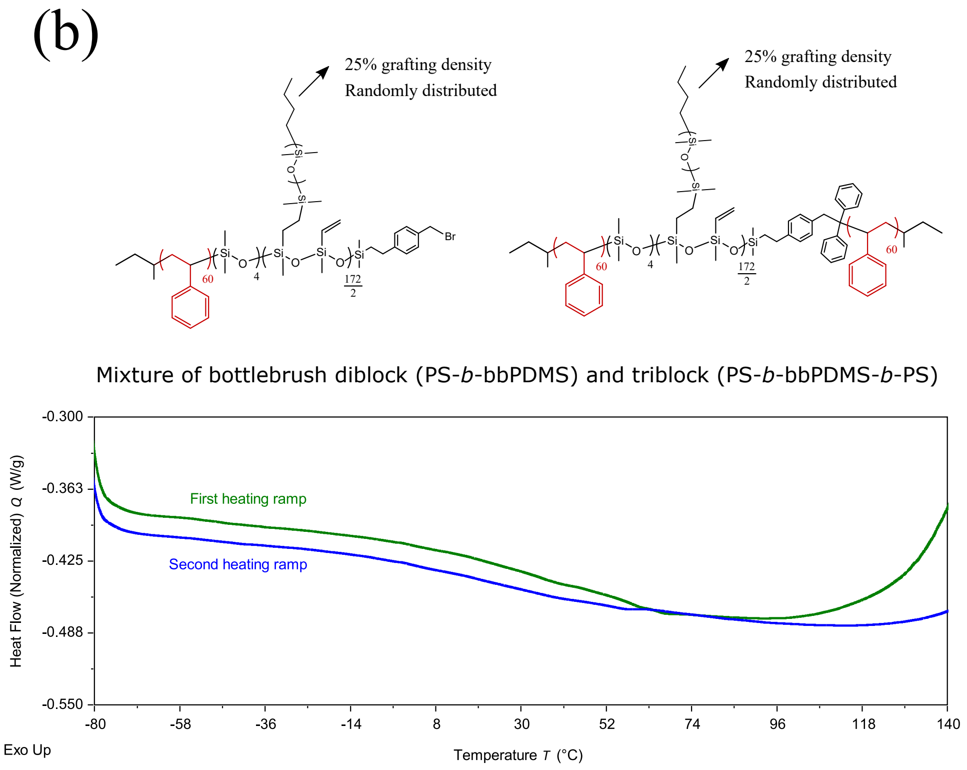

Fig. S16: DSC thermograms of **(a)** linear PS-*b*-PMVS diblock and **(b)** mixture of bottlebrush diblock (PS-*b*-bbPDMS) and triblock (PS-*b*-bbPDMS-*b*-PS) at heating rate of 10°C/min. A thermal glass transition of 73°C was found for the diblock while no transition was found for the final bottlebrush mixture due to the low mass fraction of polystyrene present in this material (≈ 6.5 polystyrene wt%).

TGA and DTG curves of linear PS-*b*-PMVS diblock and the final product which is a mixture of bottlebrush diblock (PS-*b*-bbPDMS) and bottlebrush triblock (PS-*b*-bbPDMS-*b*-PS) are compared in Fig. S17. Both samples exhibit the first main decomposition step at approximately 410°C in line with the carbonaceous decomposition with the maximum decomposition temperature (T_{max}) of 435 and 450°C for the linear diblock and the bottlebrush mixture, respectively. The bottlebrush mixture shows a second rapid decomposition step with T_{max} of 573°C which can be mainly ascribed to the decomposition of Si−O bonds, considering that the sample mass mostly comes from the grafted PDMS side chain, ending with the residual mass of 16%. However, the linear PS-*b*-PMVS diblock does not go through a rapid second step of decomposition. This is probably because of the high density of the vinyl groups in the sample which lead to crosslinking at high temperatures. The final residual mass for linear diblock is 59%. The delaying effect of crosslinking on the degradation behavior of the PMVS has been reported in the literature.[1,2]

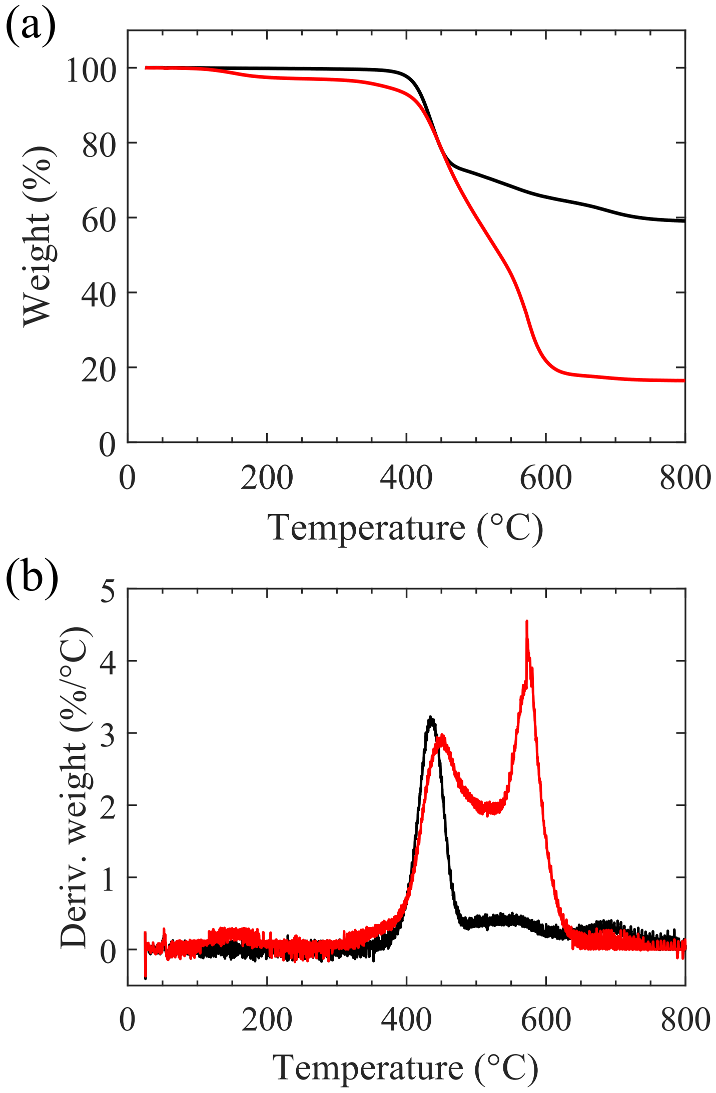

Fig. S17: Thermal degradation behavior. **(a)** TGA and **(b)** DTG curves of (—) linear PS-*b*-PMVS diblock and (—) mixture of bottlebrush diblock (PS-*b*-bbPDMS) and triblock (PS-*b*-bbPDMS-*b*-PS) under inert atmosphere at heating rate of 5°C/min.

References

(1) Ambadas, G.; Packirisamy, S.; Radhakrishnan, T. S.; Ninan, K. N. Synthesis, characterization, and thermal properties of poly(methylvinylsilylene-co-styrene). *Journal of Applied Polymer Science* **2004**, *91*, 3774–3784, DOI: `10.1002/app.13556`.

(2) Devapal, D.; Packirisamy, S.; Ambadas, G.; Radhakrishnan, T. S.; Krishnan, K.; Ninan, K. N. Thermal degradation kinetics of poly(methylvinylsilylene-co-styrene). *Thermochimica Acta* **2004**, *409*, 151–156, DOI: `10.1016/S0040-6031(03)00355-1`.
