# Supplementary figures and images for: Synthesis of well-defined linear–bottlebrush–linear triblock copolymer towards architecturally-tunable soft materials
Source: Polym Chem. 2022 Jul 20;13(32):4666–74. doi: 10.1039/d2py00841f (PMC9379773; doi:10.1039/d2py00841f)

Linear PS-*b*-PMVS diblock

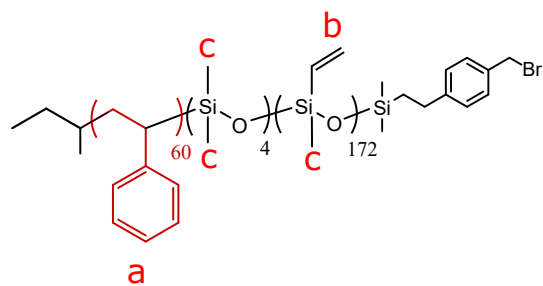

Linear PS-*b*-PMVS-*b*-PS triblock

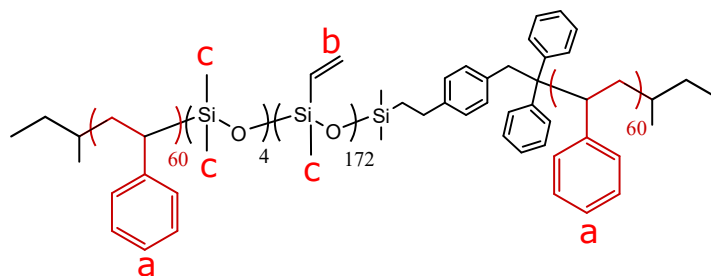

Excess PS

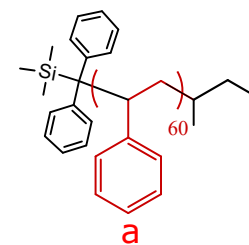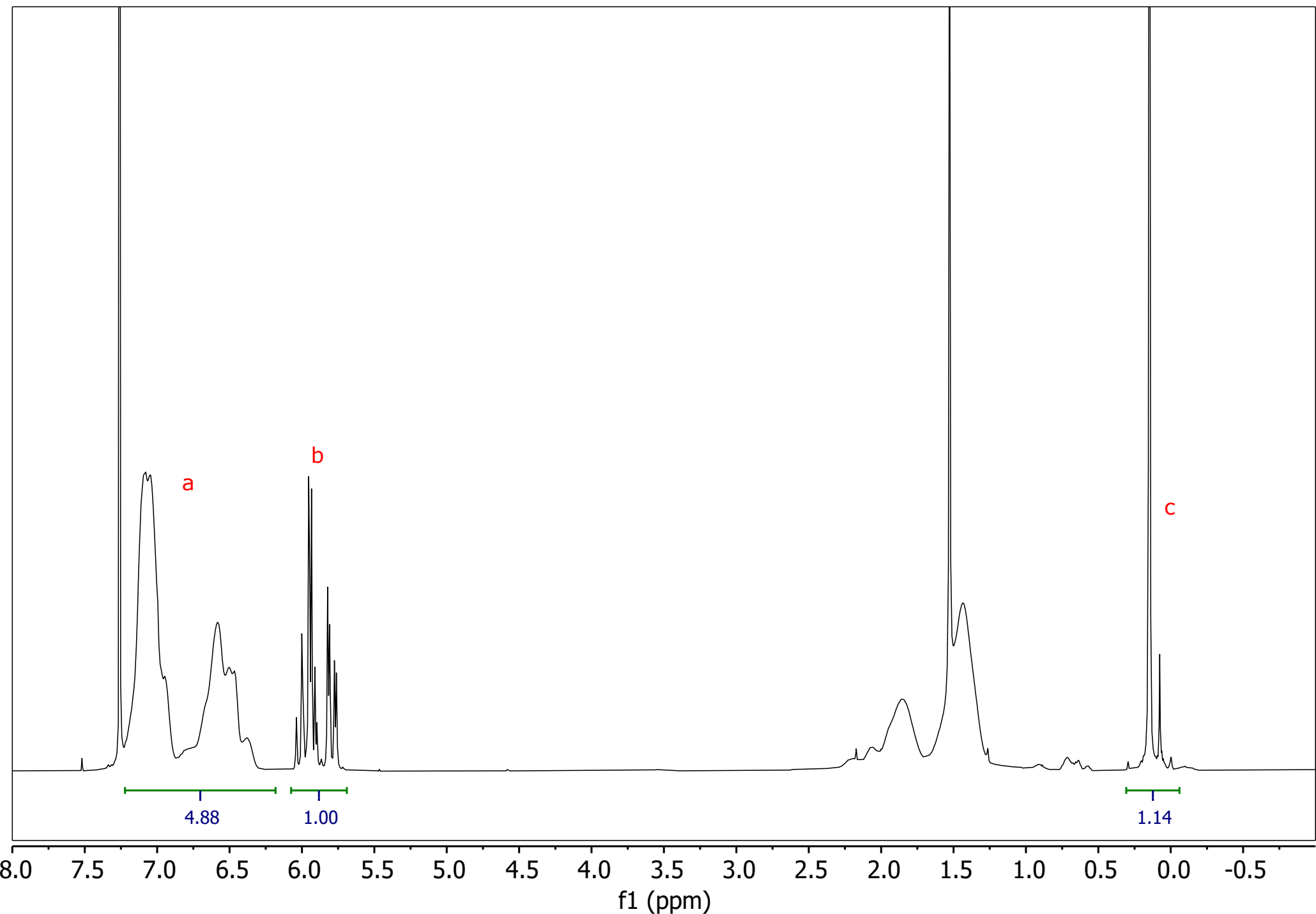

Supplement: PY-013-D2PY00841F-s002 [file PY-013-D2PY00841F-s002.pdf]

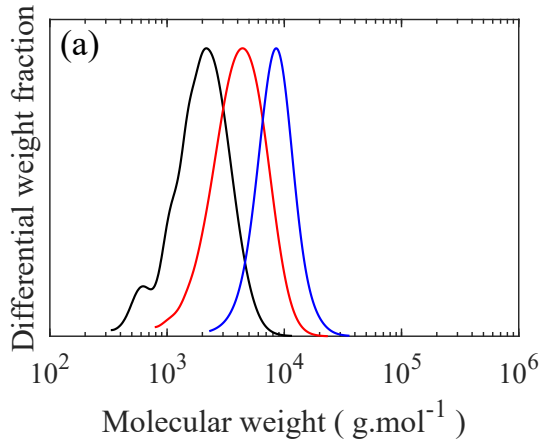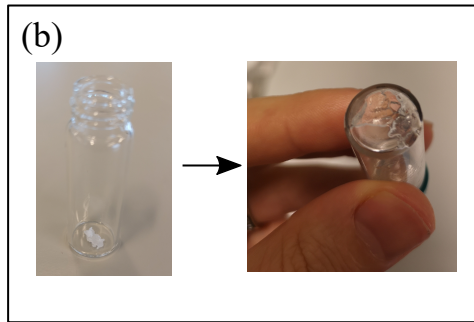

Supplement: PY-013-D2PY00841F-s003 [file PY-013-D2PY00841F-s003.pdf]

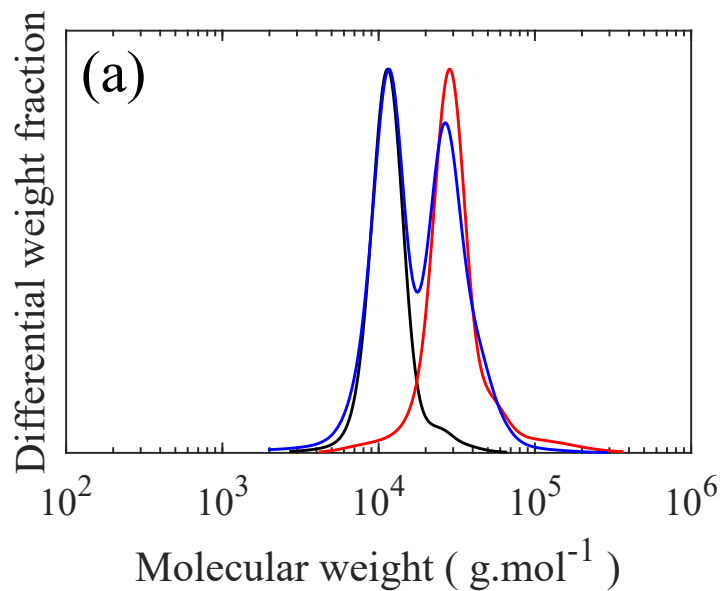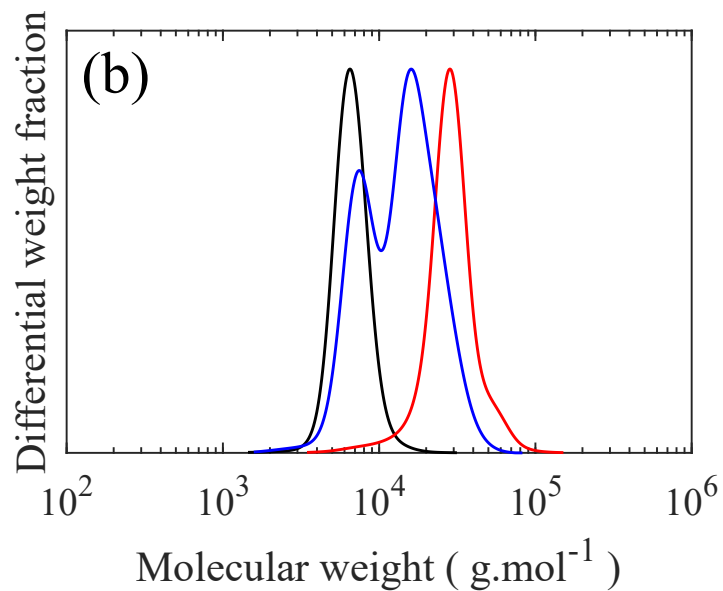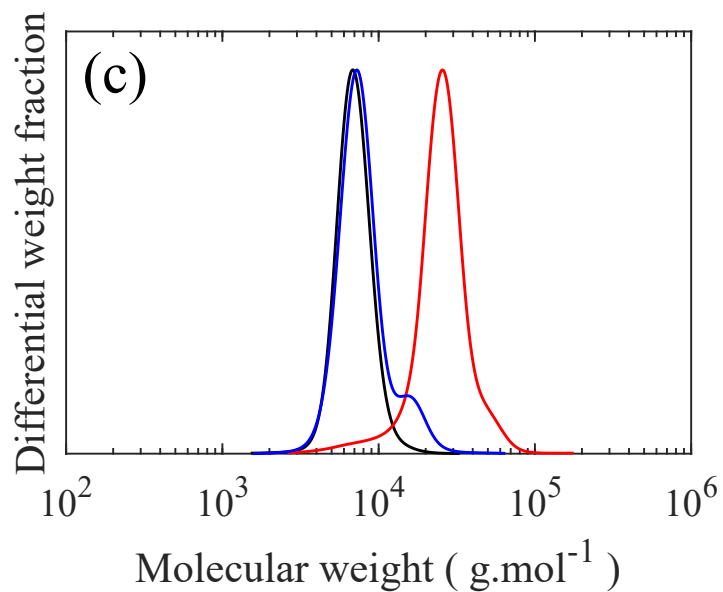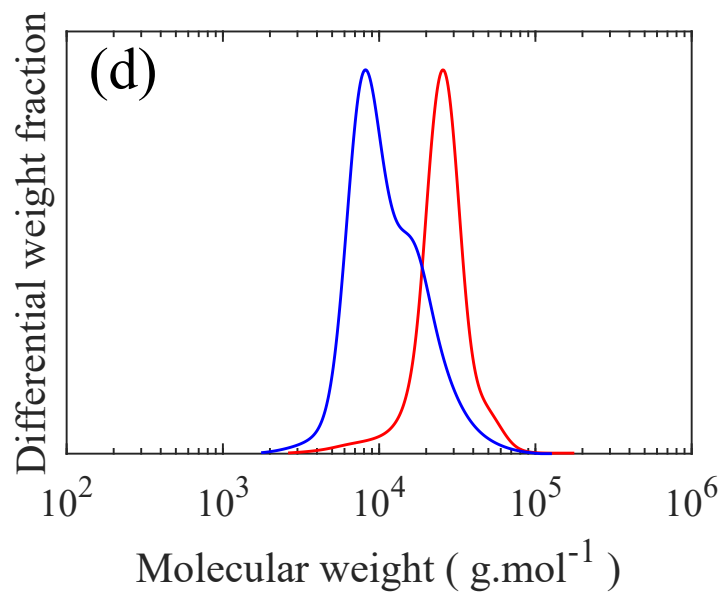

Supplement: PY-013-D2PY00841F-s004 [file PY-013-D2PY00841F-s004.pdf]

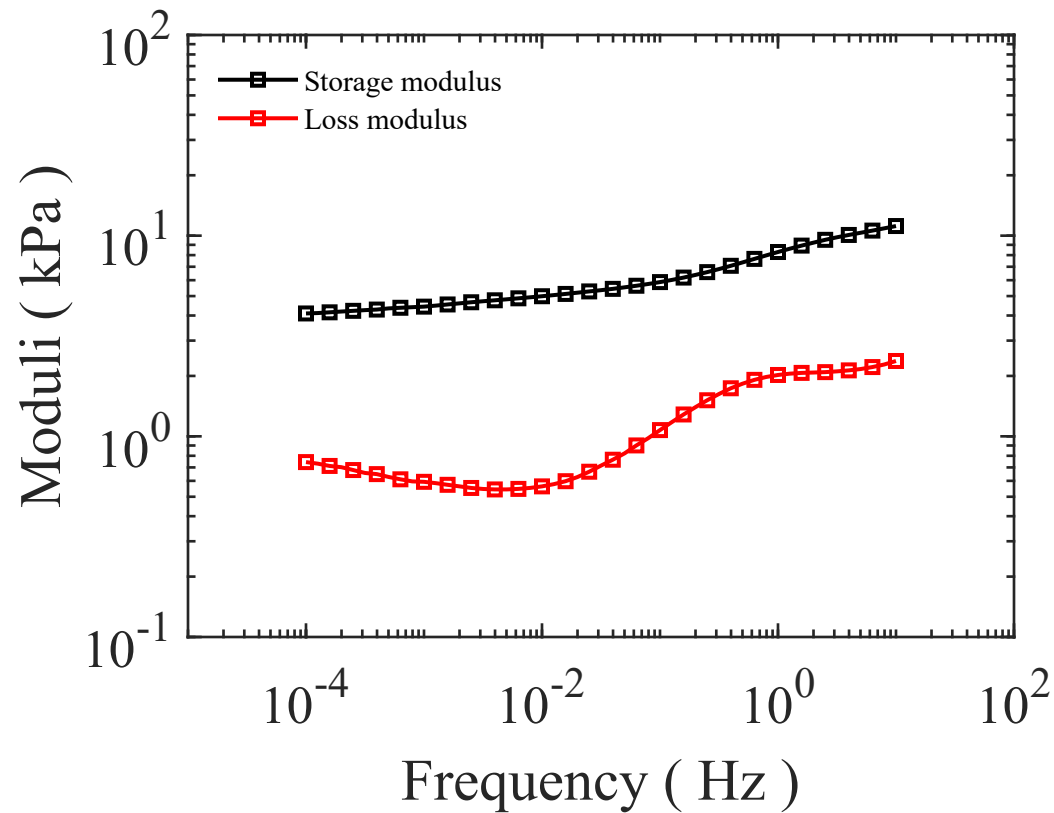

Supplement: PY-013-D2PY00841F-s006 [file PY-013-D2PY00841F-s006.pdf]

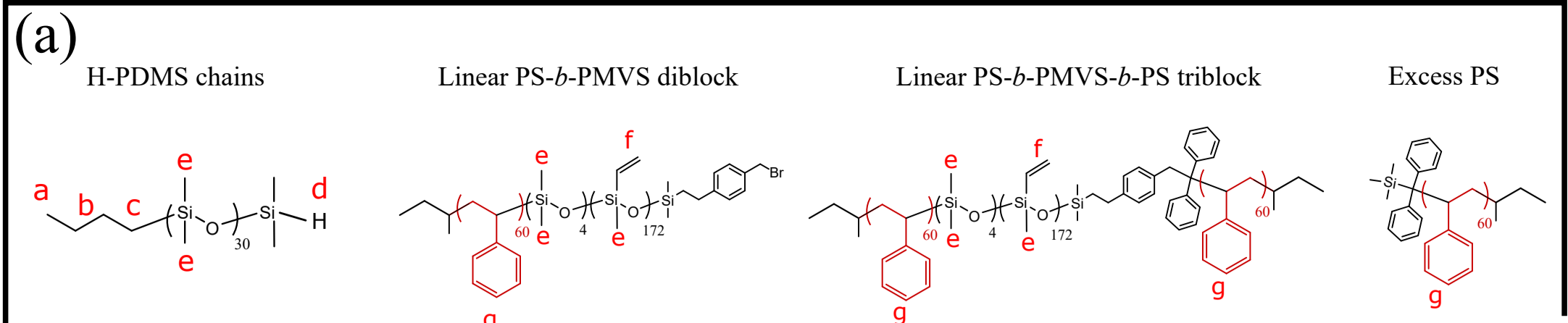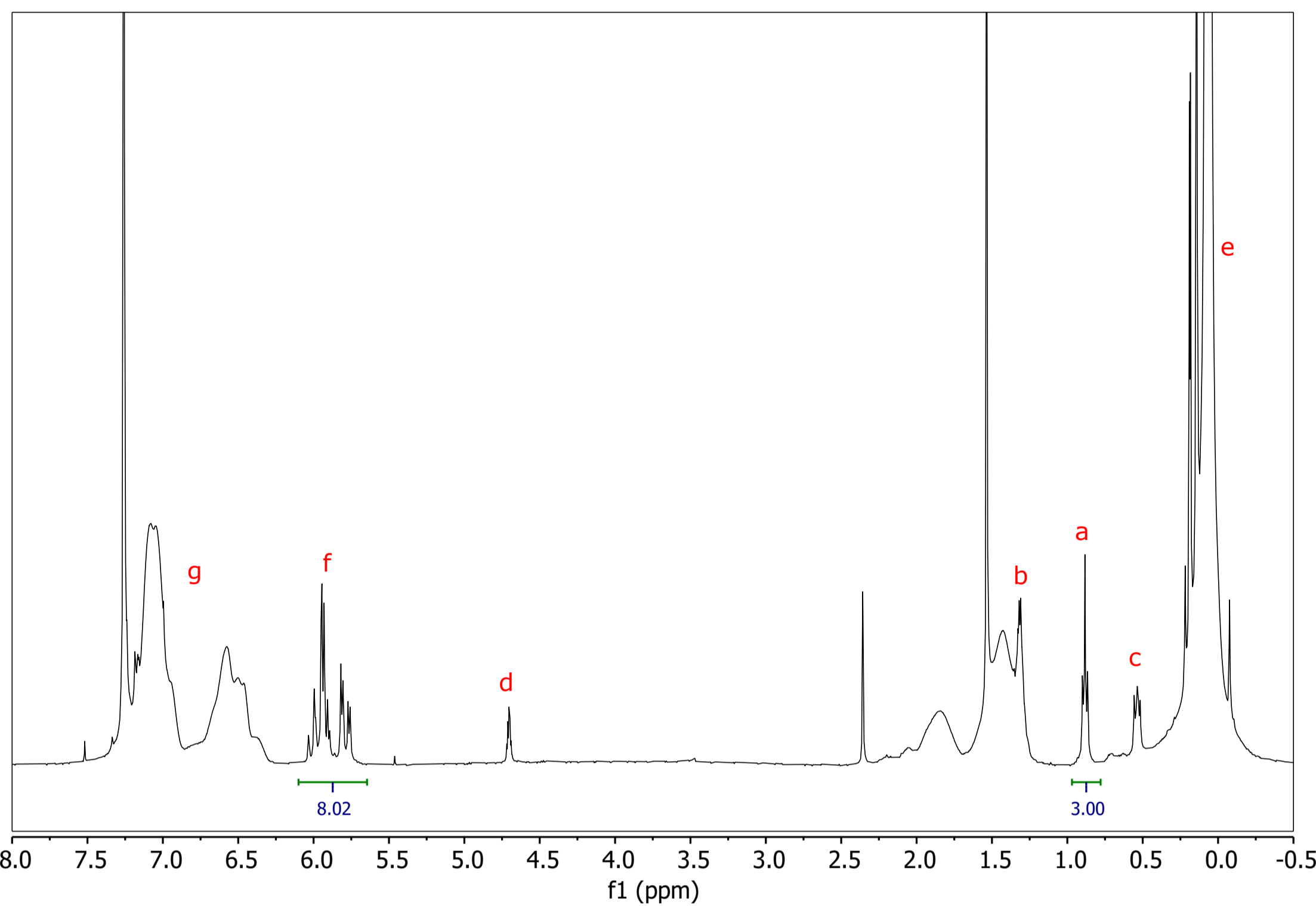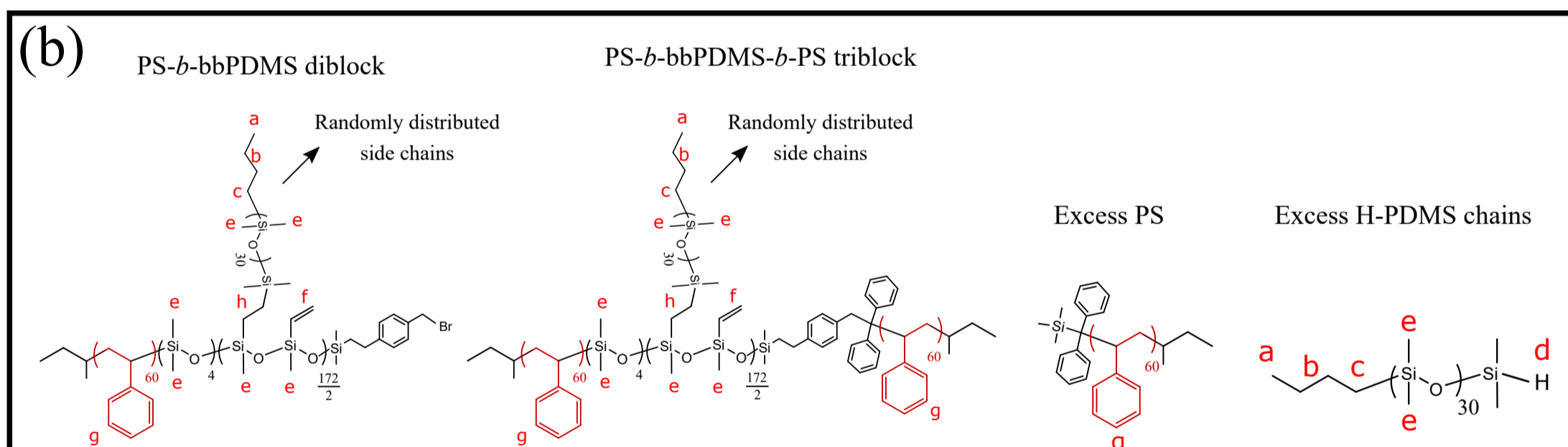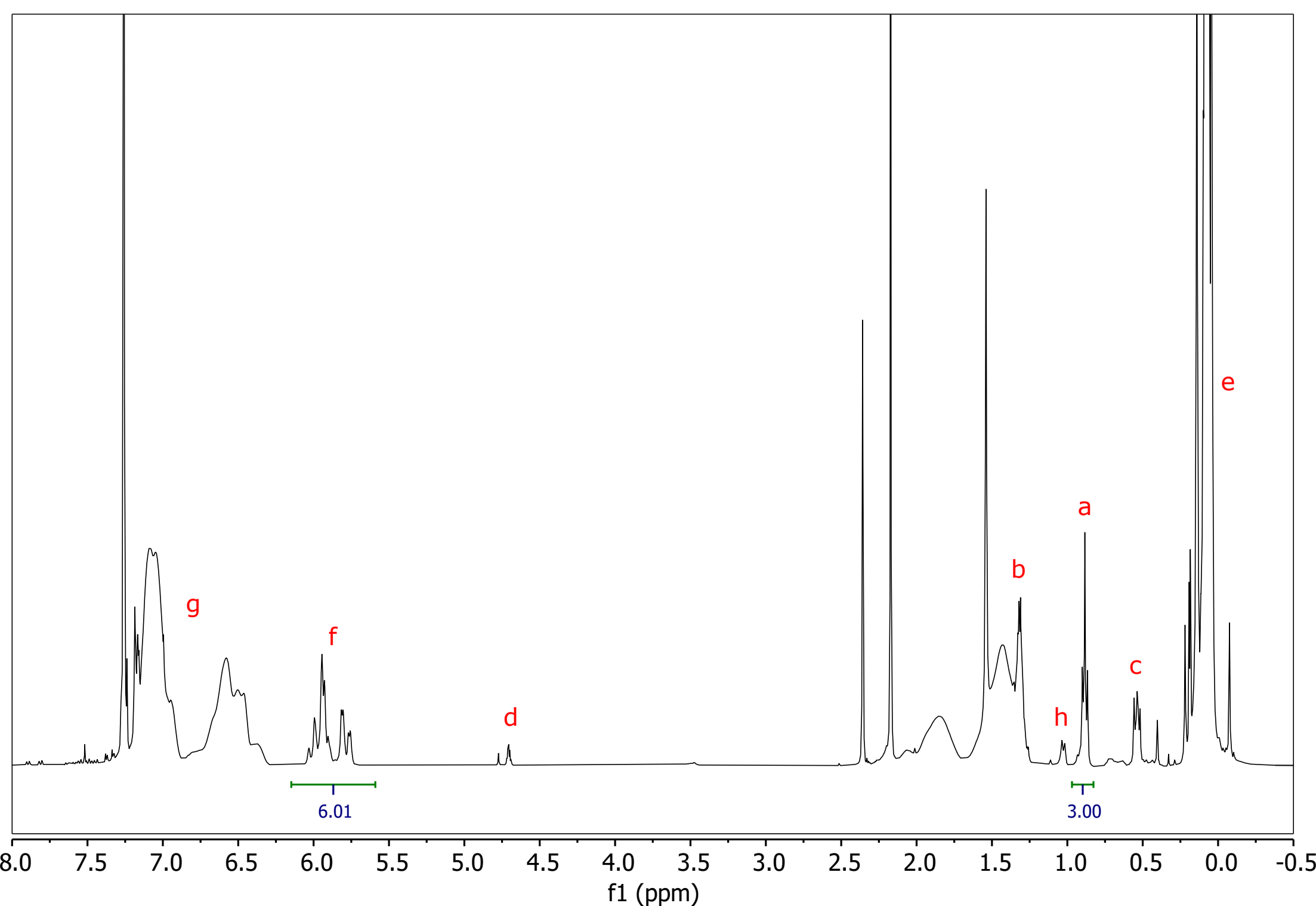

Supplement: PY-013-D2PY00841F-s007 [file PY-013-D2PY00841F-s007.pdf]

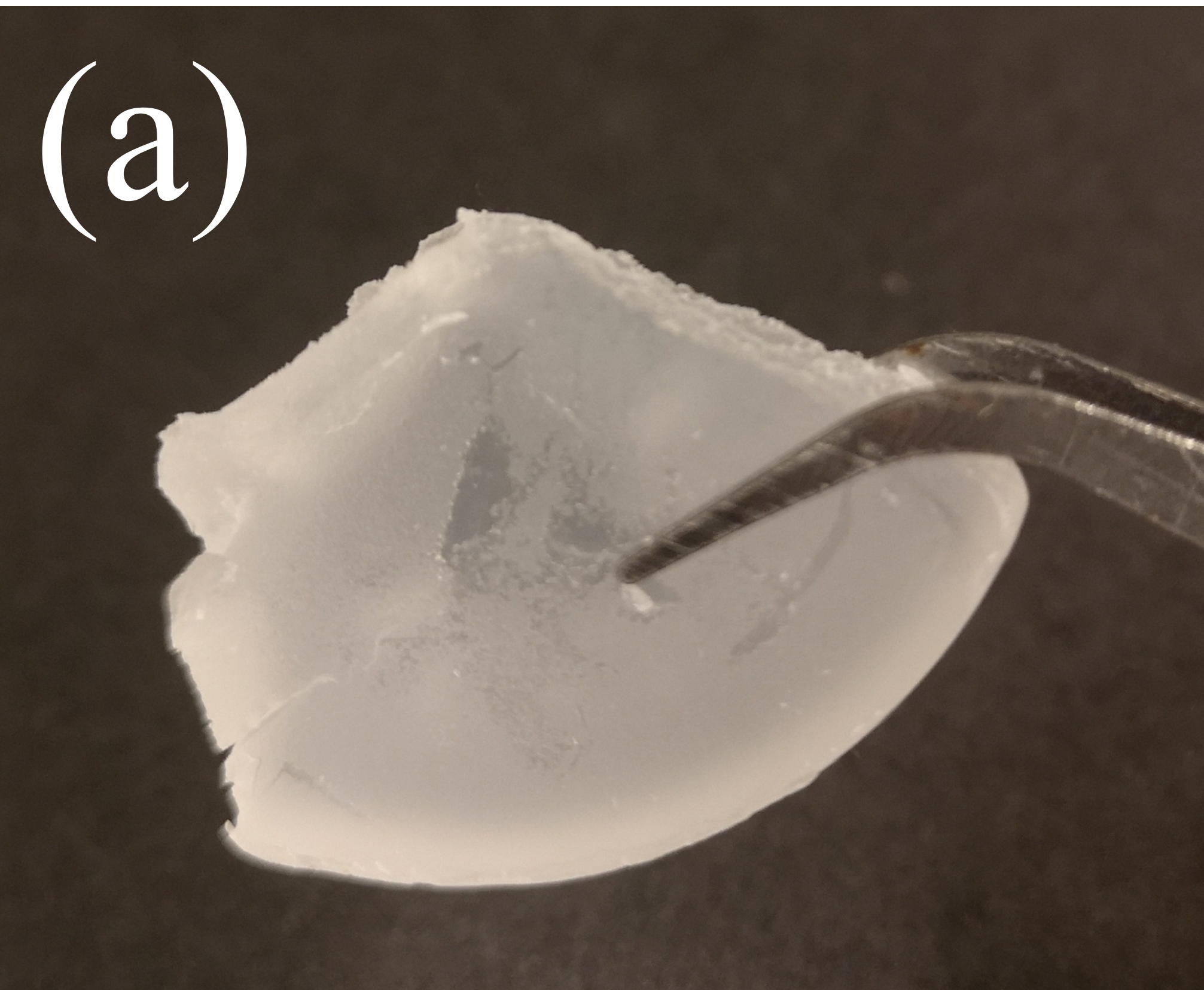

PS-*b*-PMVS

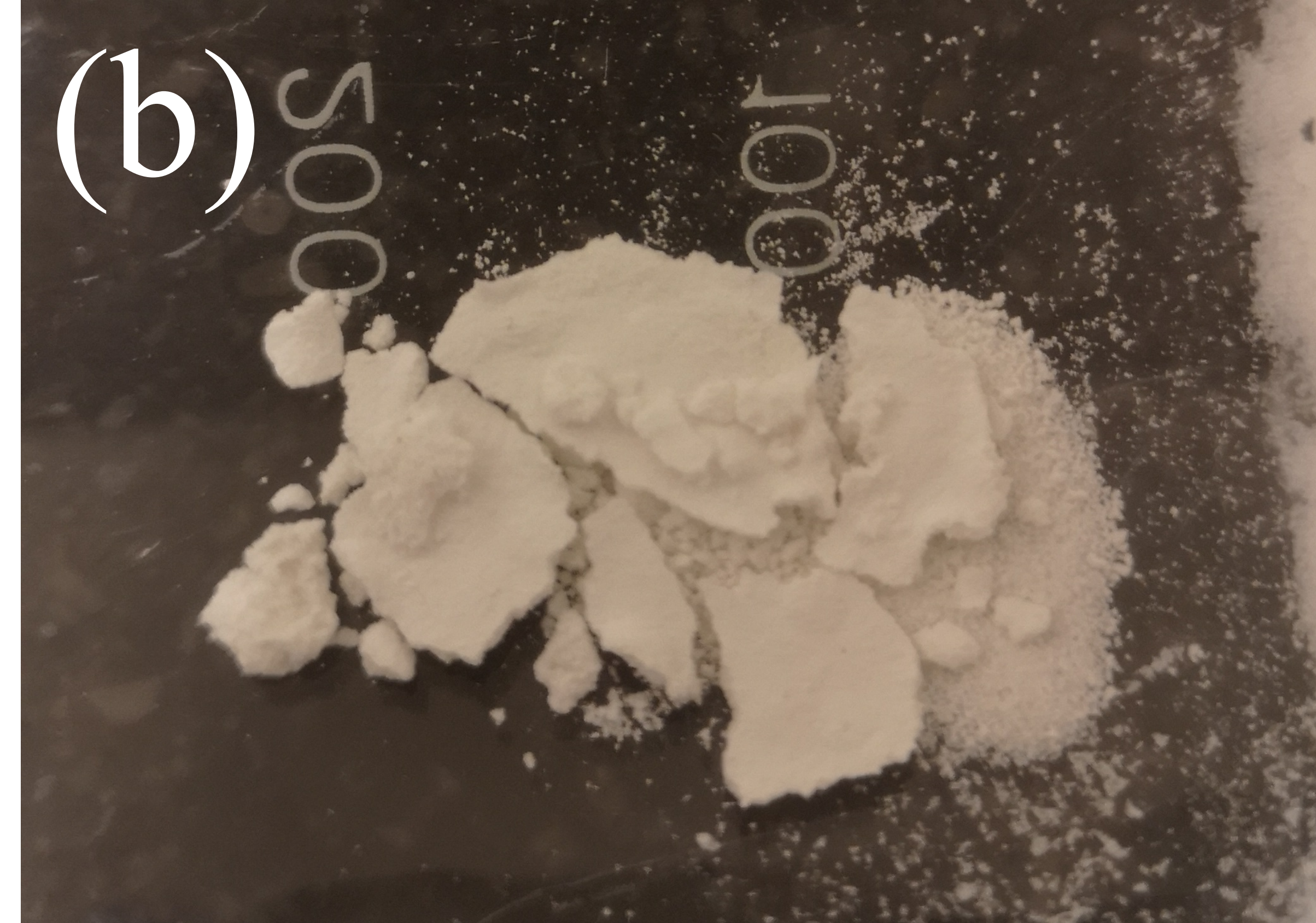

[ PS-*b*-PMVS  
PS-*b*-PMVS-*b*-PS  
PS

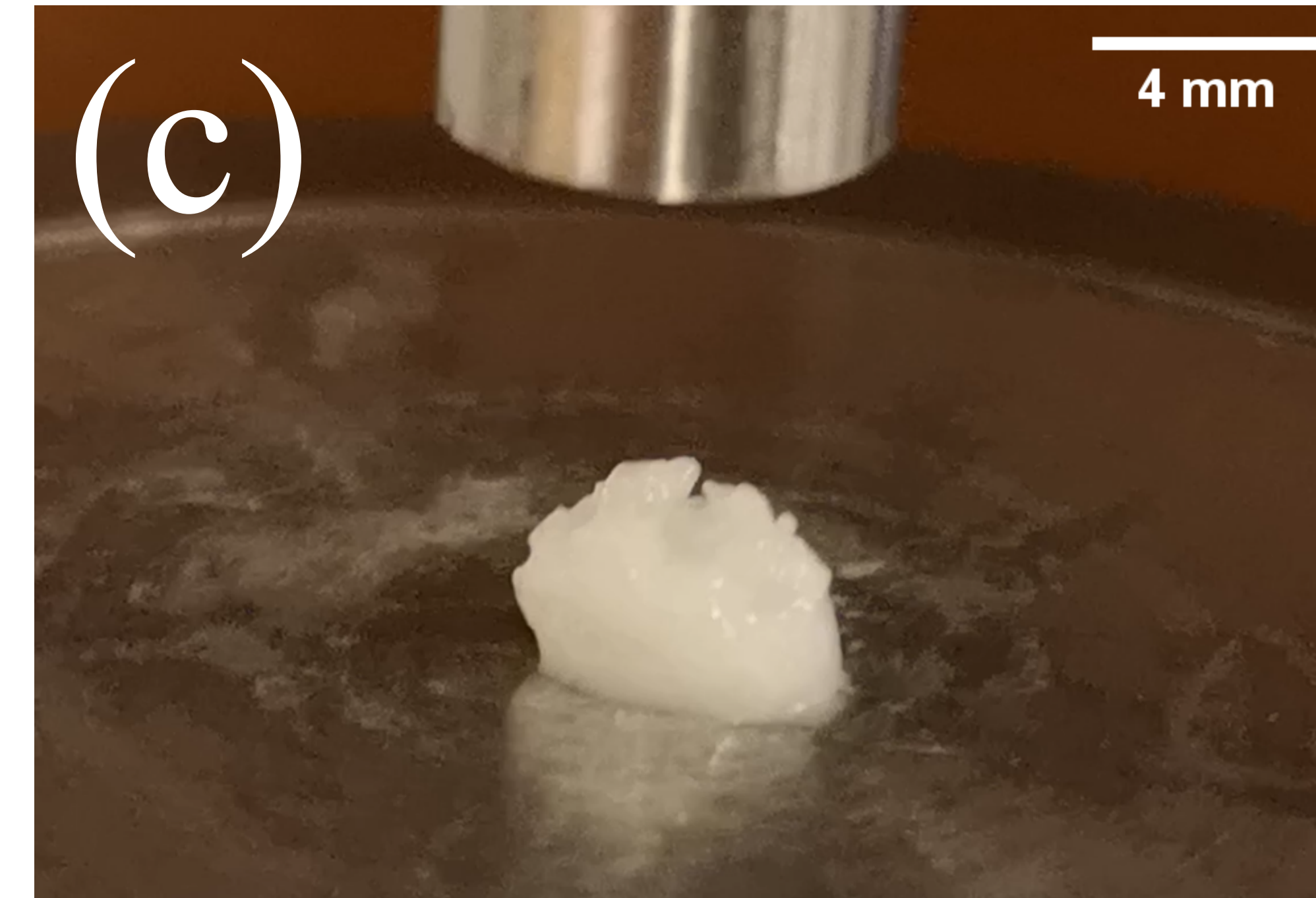

20 °C

Heating

140 °C

[ PS-*b*-bbPDMS  
PS-*b*-bbPDMS-*b*-PS

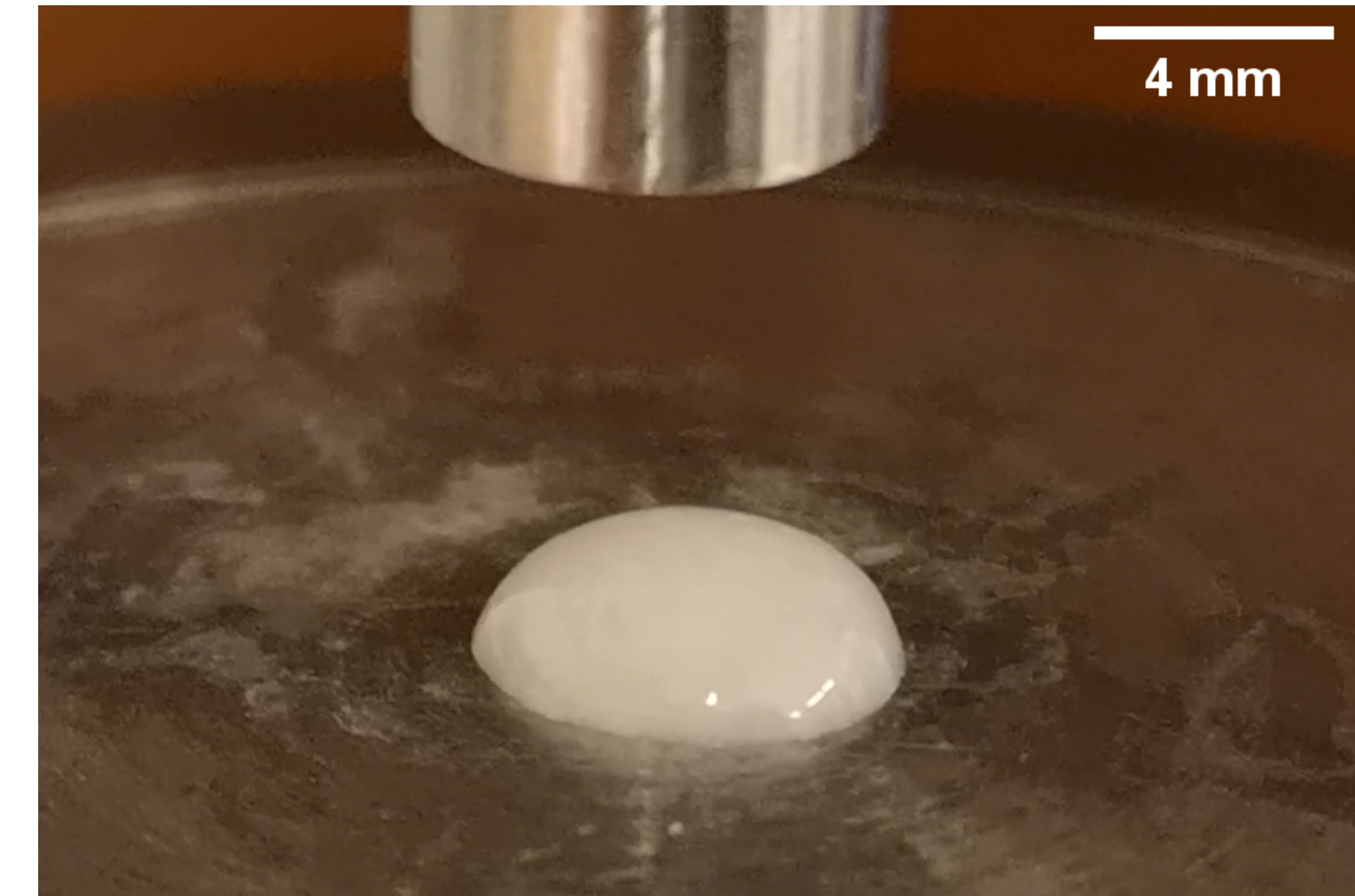

Supplement: PY-013-D2PY00841F-s009 [file PY-013-D2PY00841F-s009.pdf]

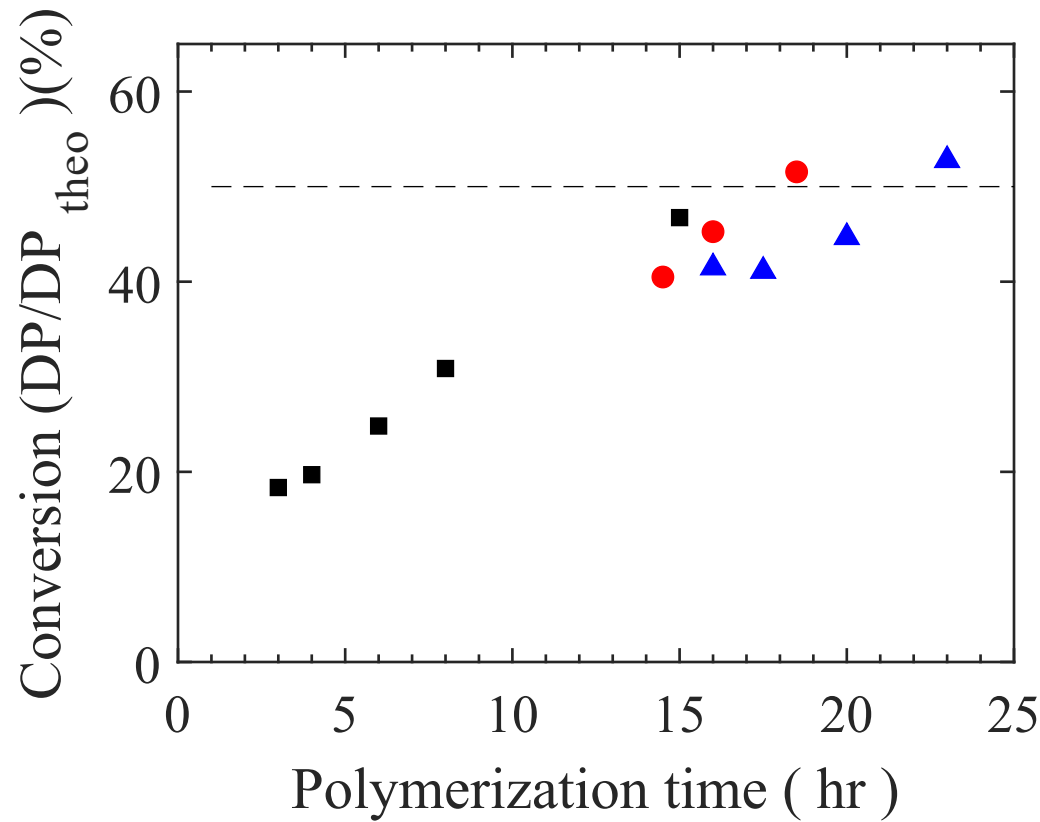

Supplement: PY-013-D2PY00841F-s010 [file PY-013-D2PY00841F-s010.pdf]

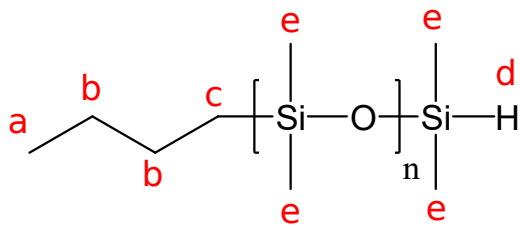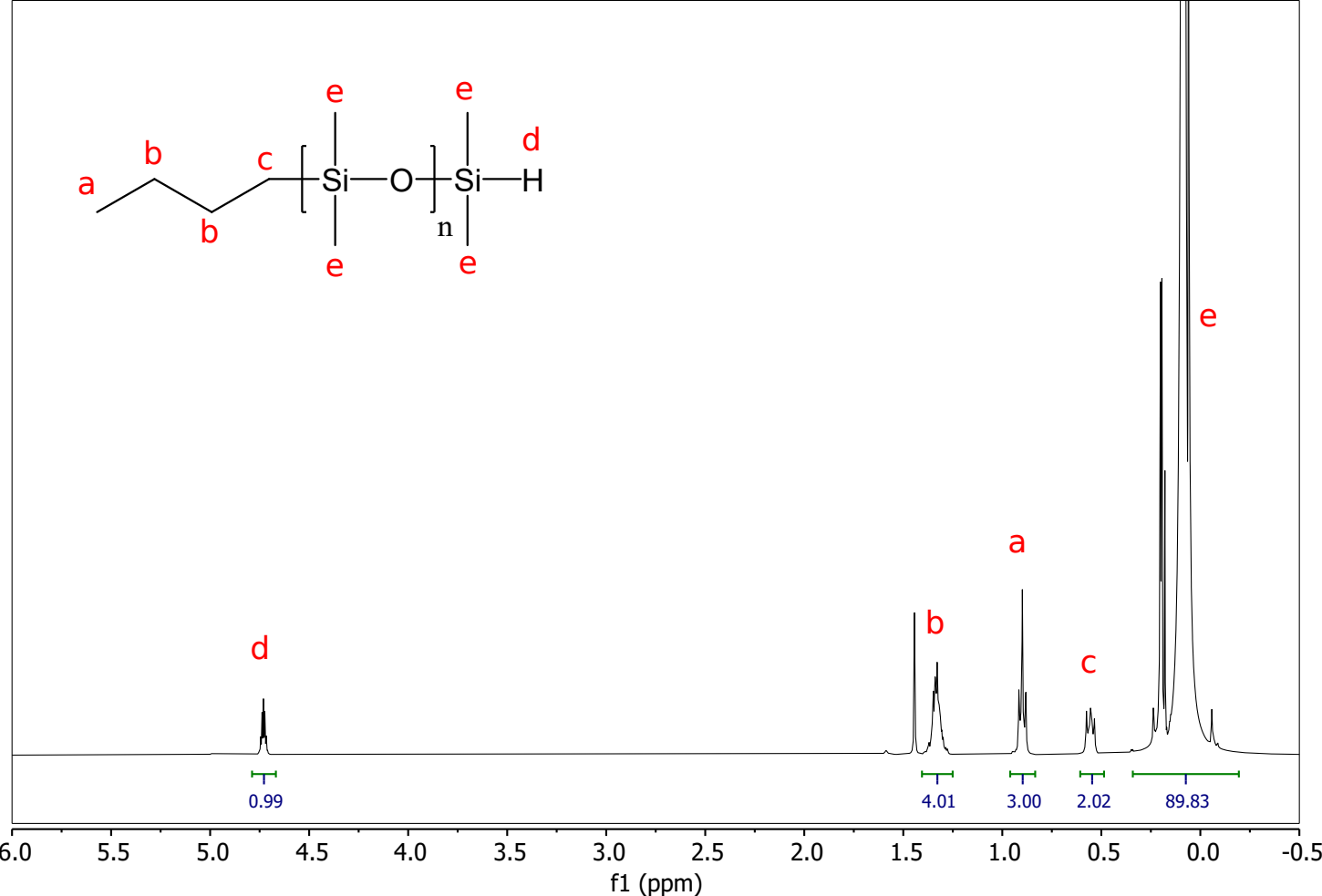

Supplement: PY-013-D2PY00841F-s011 [file PY-013-D2PY00841F-s011.pdf]

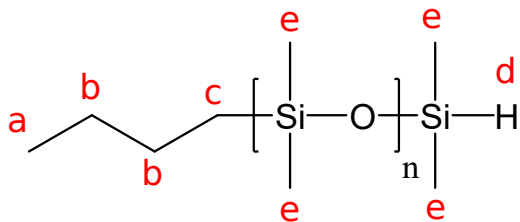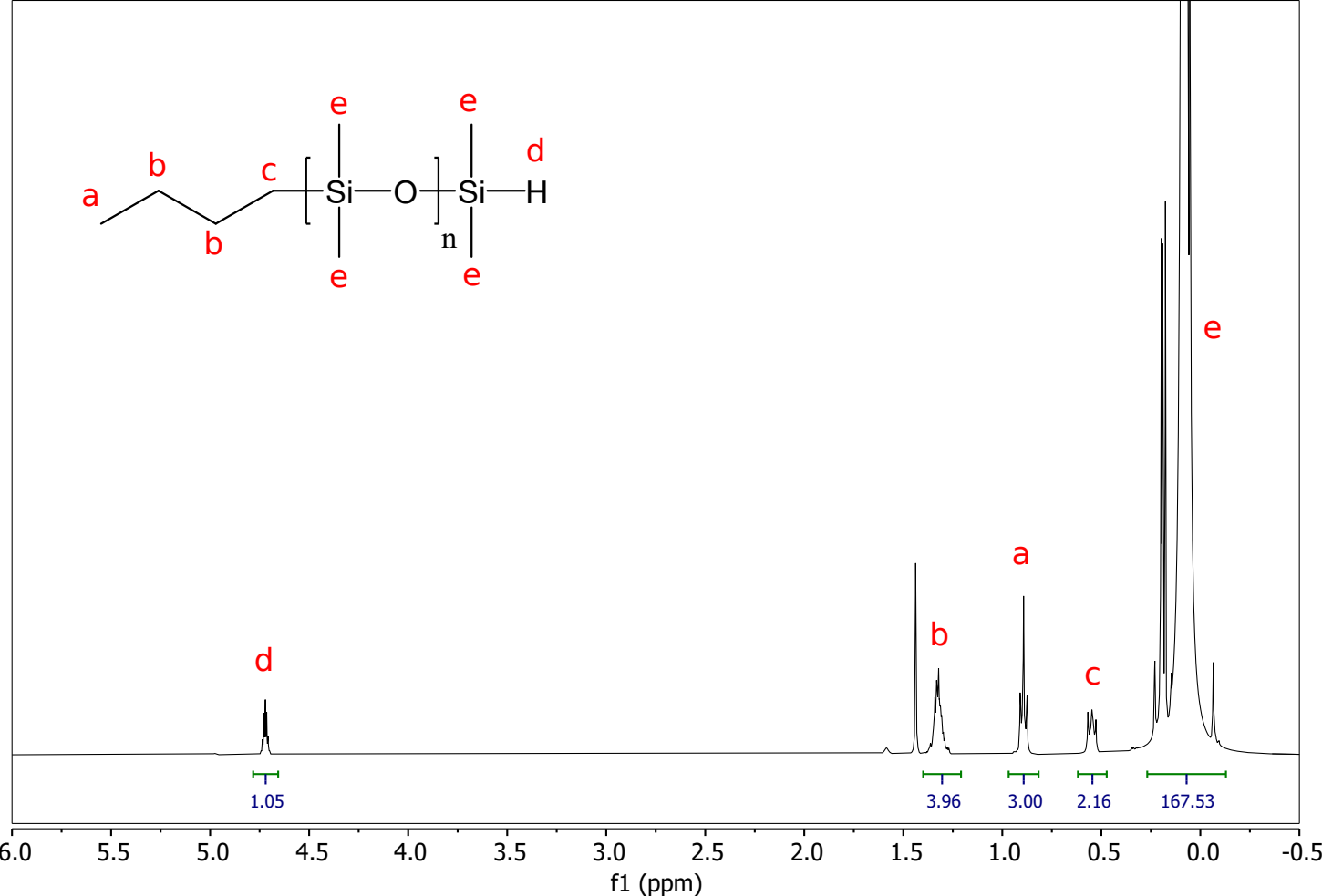

Supplement: PY-013-D2PY00841F-s012 [file PY-013-D2PY00841F-s012.pdf]

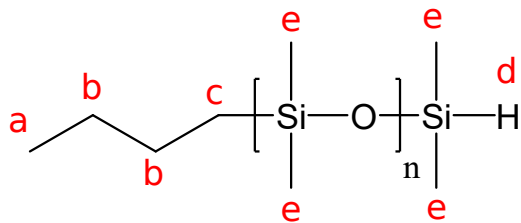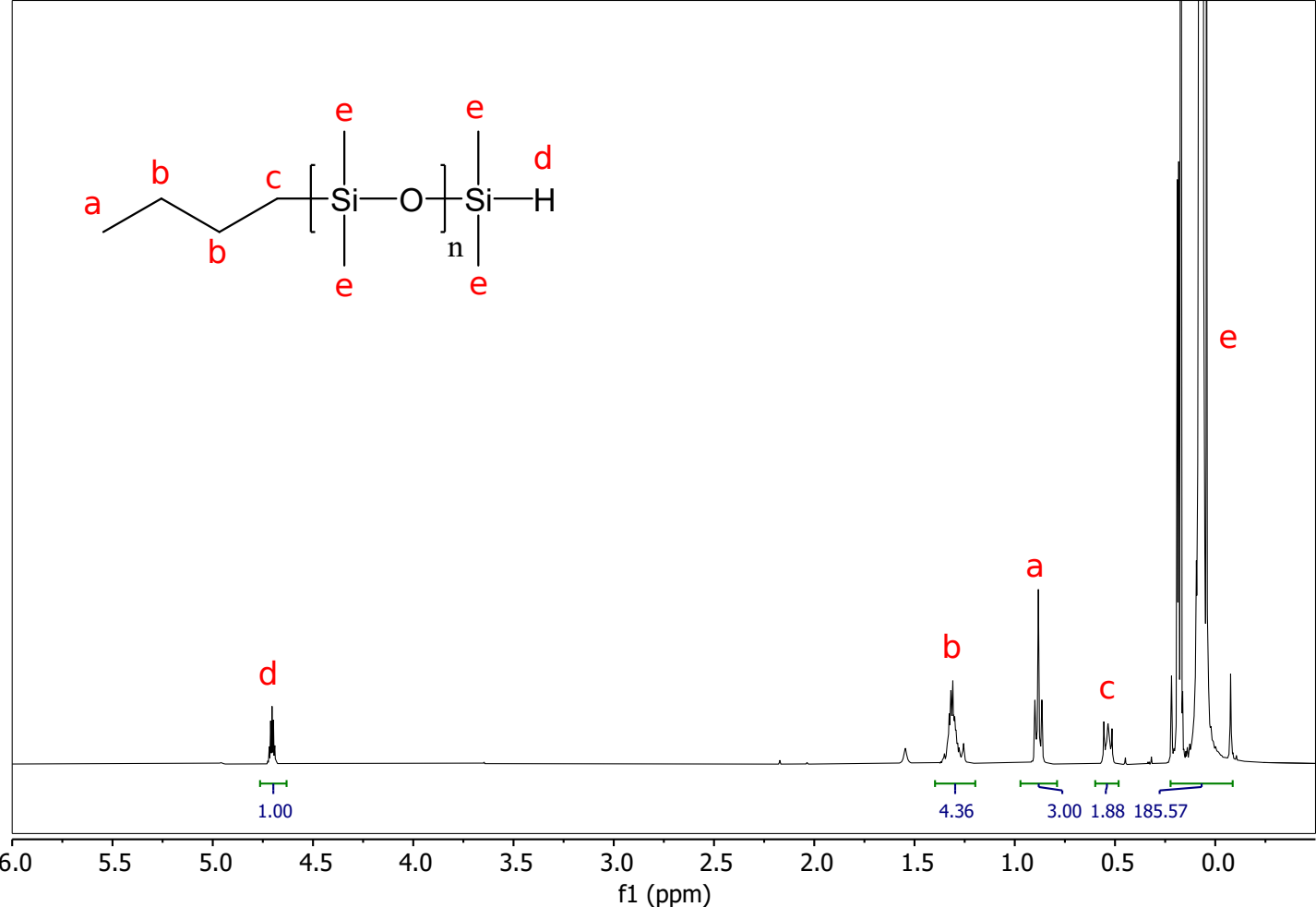

Supplement: PY-013-D2PY00841F-s013 [file PY-013-D2PY00841F-s013.pdf]

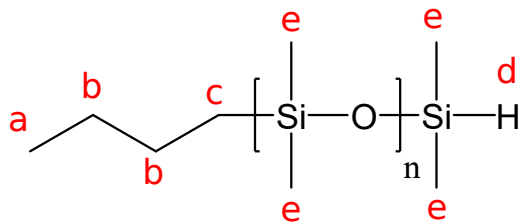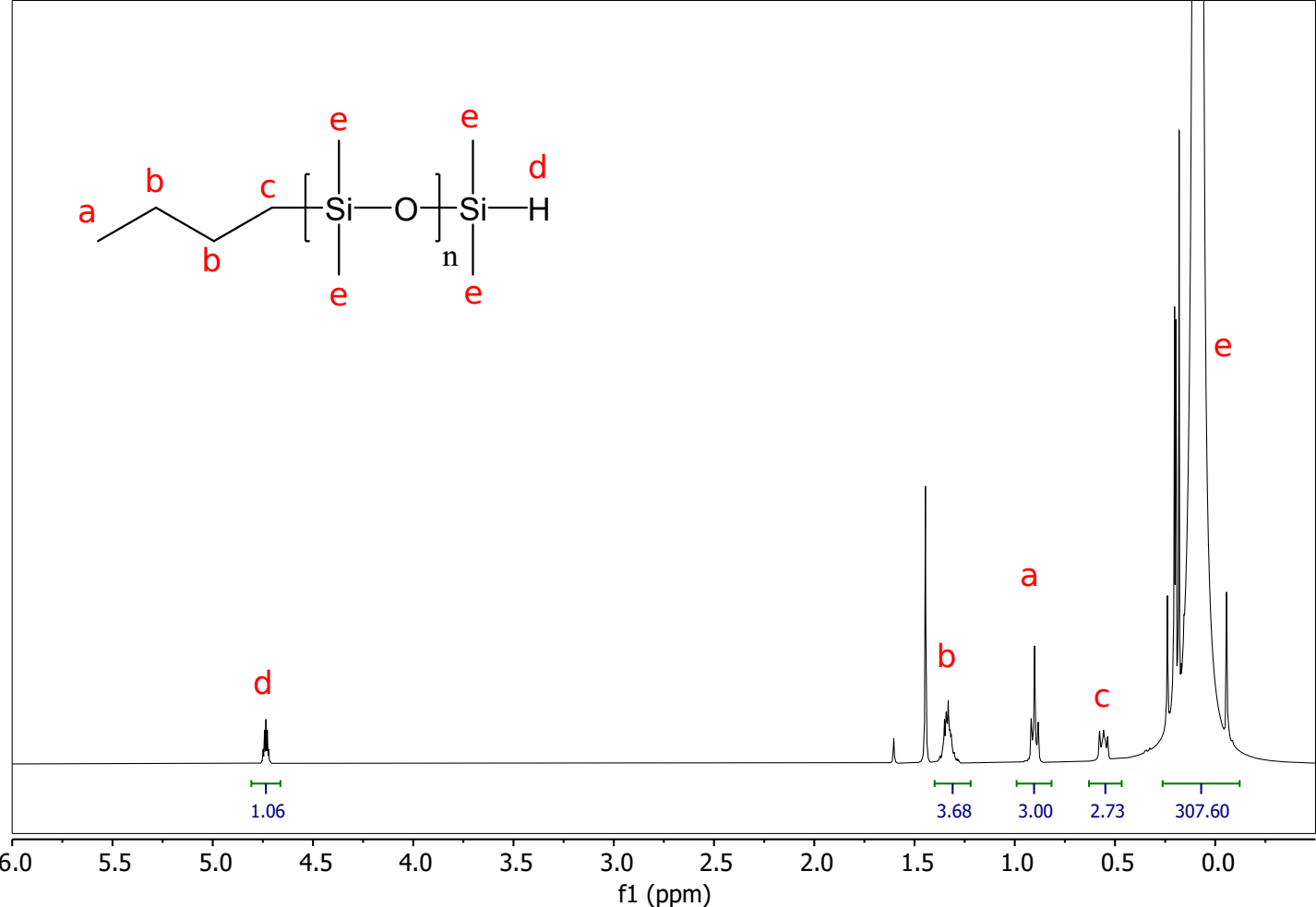

Supplement: PY-013-D2PY00841F-s014 [file PY-013-D2PY00841F-s014.pdf]

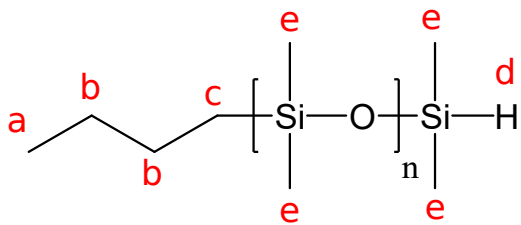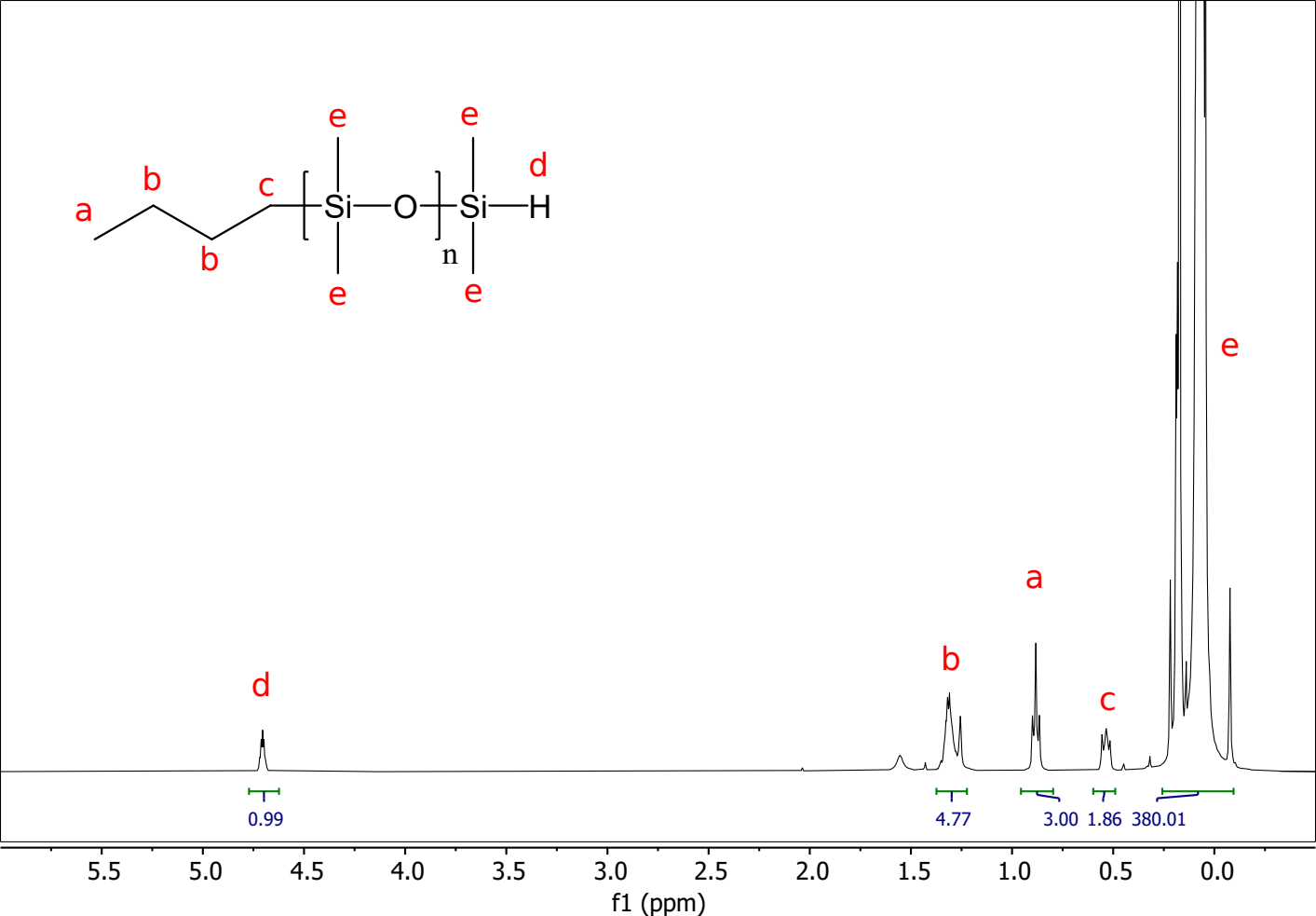

Supplement: PY-013-D2PY00841F-s015 [file PY-013-D2PY00841F-s015.pdf]

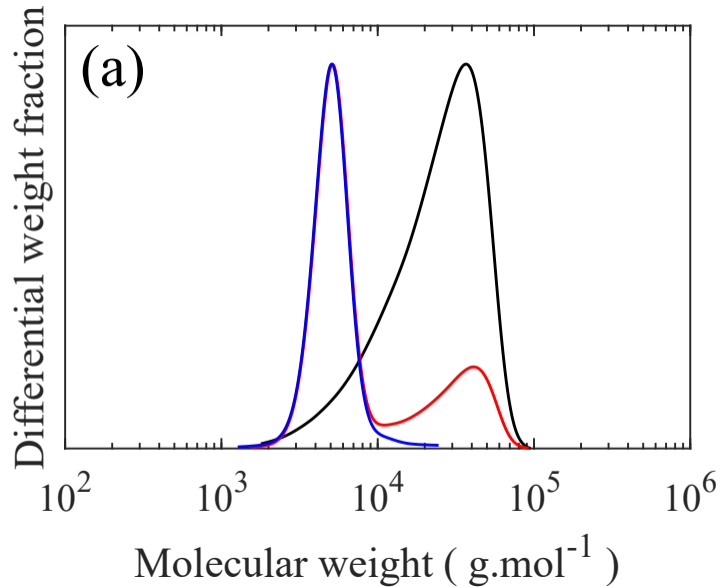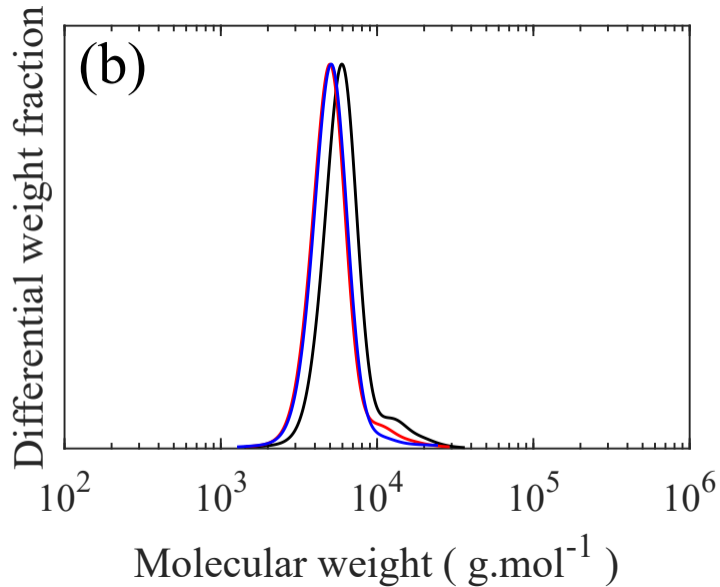

Supplement: PY-013-D2PY00841F-s016 [file PY-013-D2PY00841F-s016.pdf]

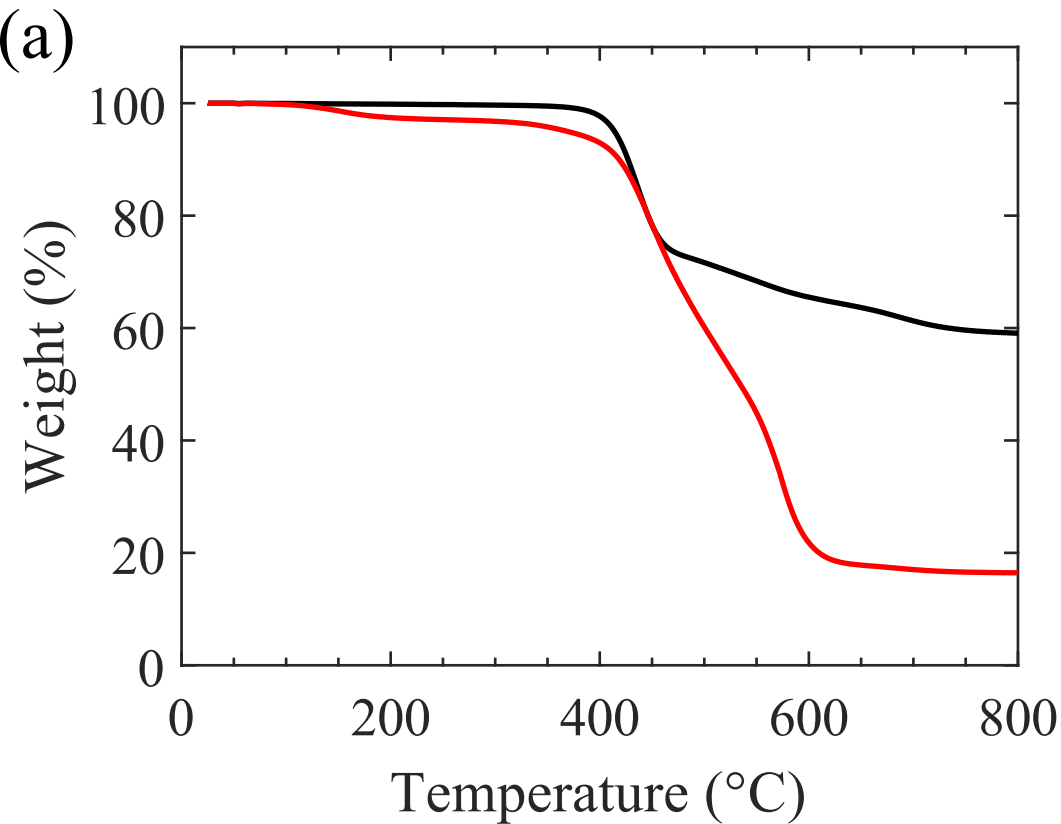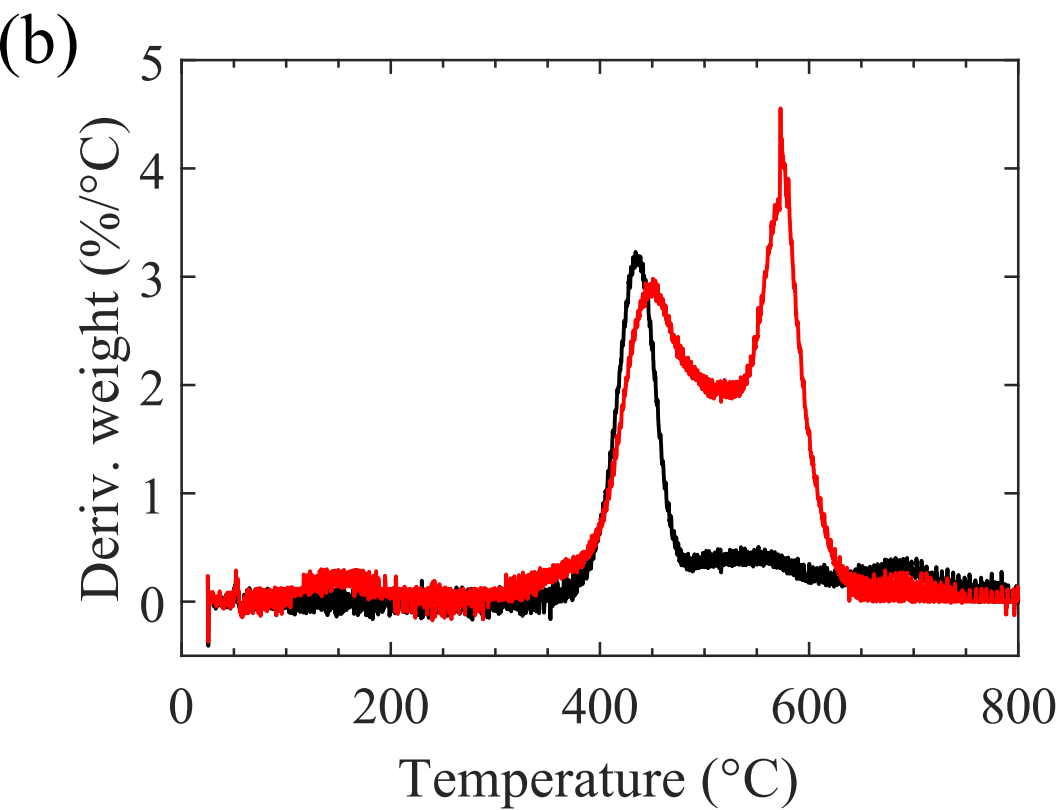

Supplement: PY-013-D2PY00841F-s017 [file PY-013-D2PY00841F-s017.pdf]

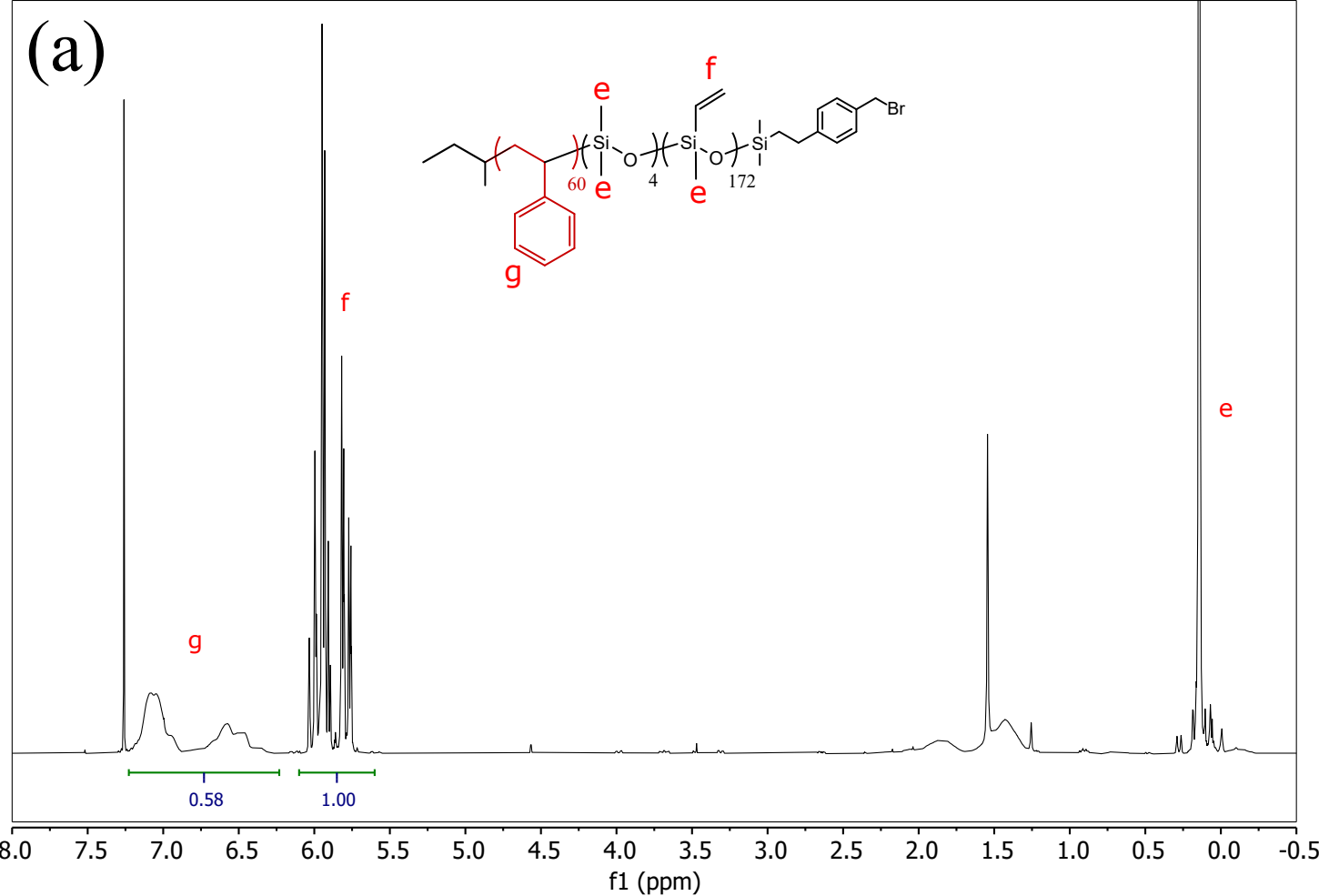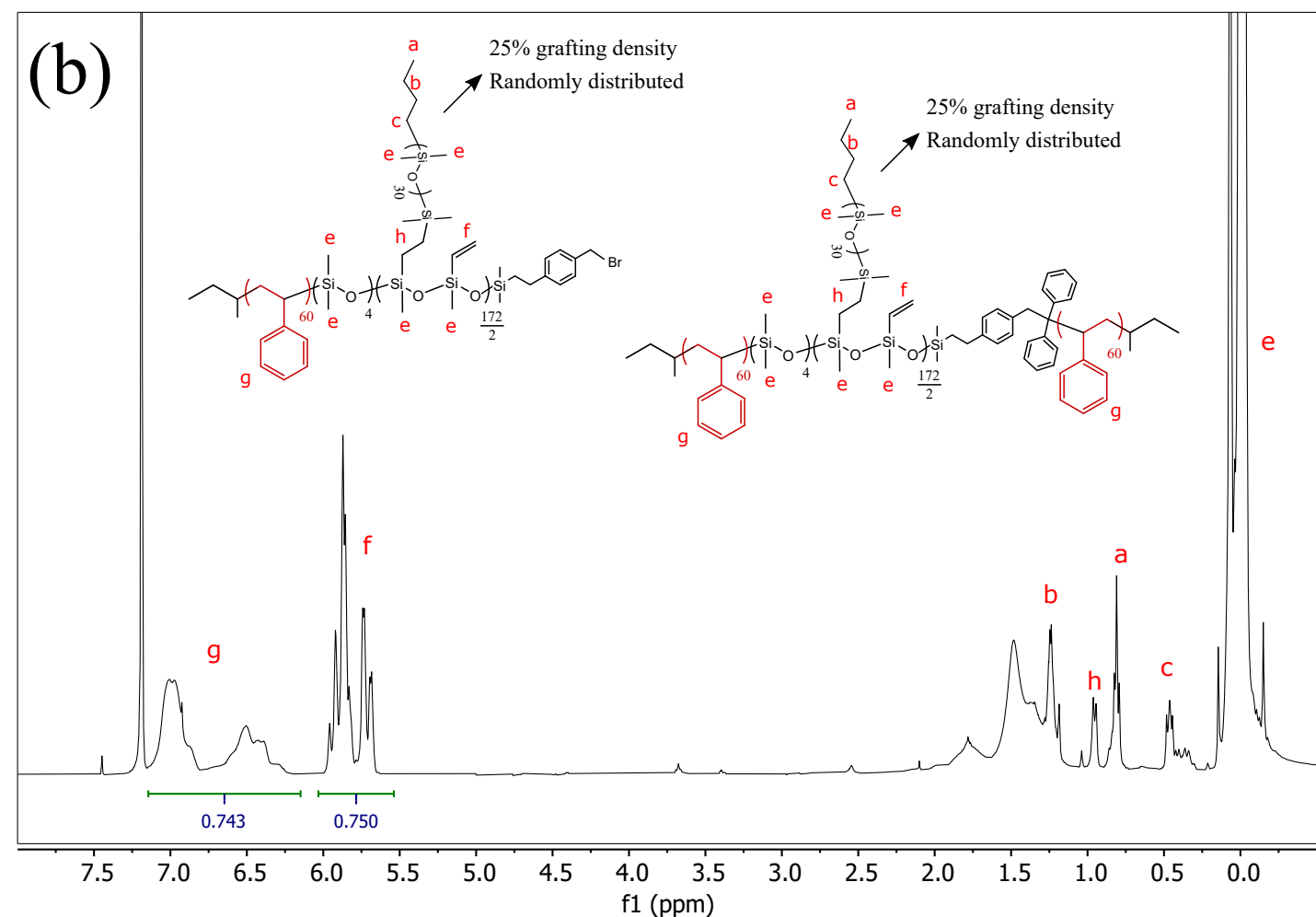

Supplement: PY-013-D2PY00841F-s018 [file PY-013-D2PY00841F-s018.pdf]
