# Supplementary material for: Synthesis of well-defined linear–bottlebrush–linear triblock copolymer towards architecturally-tunable soft materials
Source: Polym Chem. 2022 Jul 20;13(32):4666–74. doi: 10.1039/d2py00841f (PMC9379773; doi:10.1039/d2py00841f)
Supplement: PY-013-D2PY00841F-s005 [file PY-013-D2PY00841F-s005.pdf]

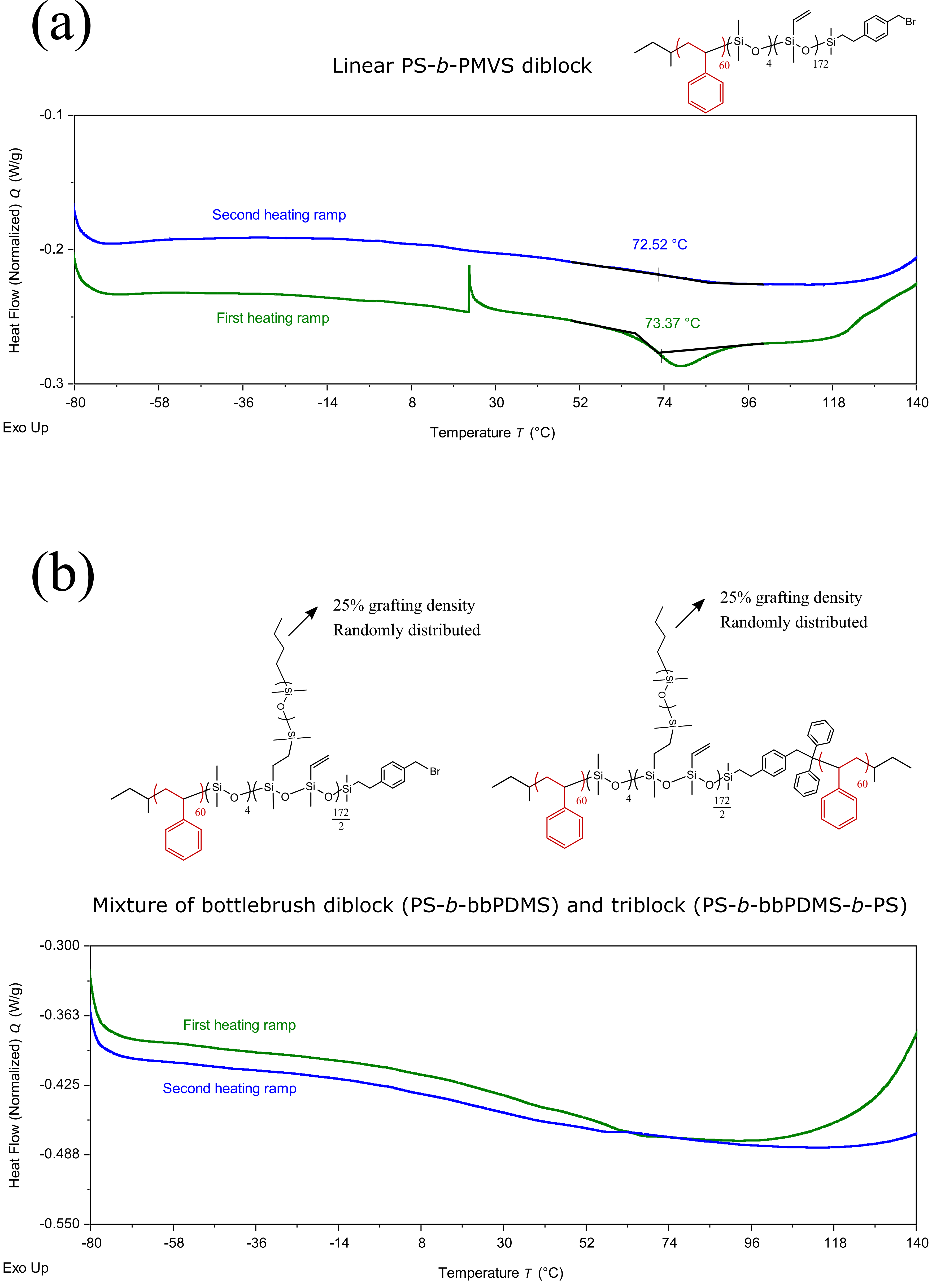
(a)
Linear PS-b-PMVS diblock
Si
O
Br
60
4
172
Heat Flow (Normalized) Q (W/g)
-0.1
-0.2
-0.3
Second heating ramp
72.52 °C
First heating ramp
73.37 °C
-80
-58
-36
-14
8
30
52
74
96
118
140
Exo Up
Temperature T (°C)
(b)
25% grafting density
Randomly distributed
25% grafting density
Randomly distributed
Si
O
Br
60
4
172
2
Mixture of bottlebrush diblock (PS-b-bbPDMS) and triblock (PS-b-bbPDMS-b-PS)
Heat Flow (Normalized) Q (W/g)
-0.300
-0.363
-0.425
-0.488
-0.550
First heating ramp
Second heating ramp
-80
-58
-36
-14
8
30
52
74
96
118
140
Exo Up
Temperature T (°C)
