# Supplementary material for: Synthesis of well-defined linear–bottlebrush–linear triblock copolymer towards architecturally-tunable soft materials
Source: Polym Chem. 2022 Jul 20;13(32):4666–74. doi: 10.1039/d2py00841f (PMC9379773; doi:10.1039/d2py00841f)
Supplement: PY-013-D2PY00841F-s008 [file PY-013-D2PY00841F-s008.pdf]

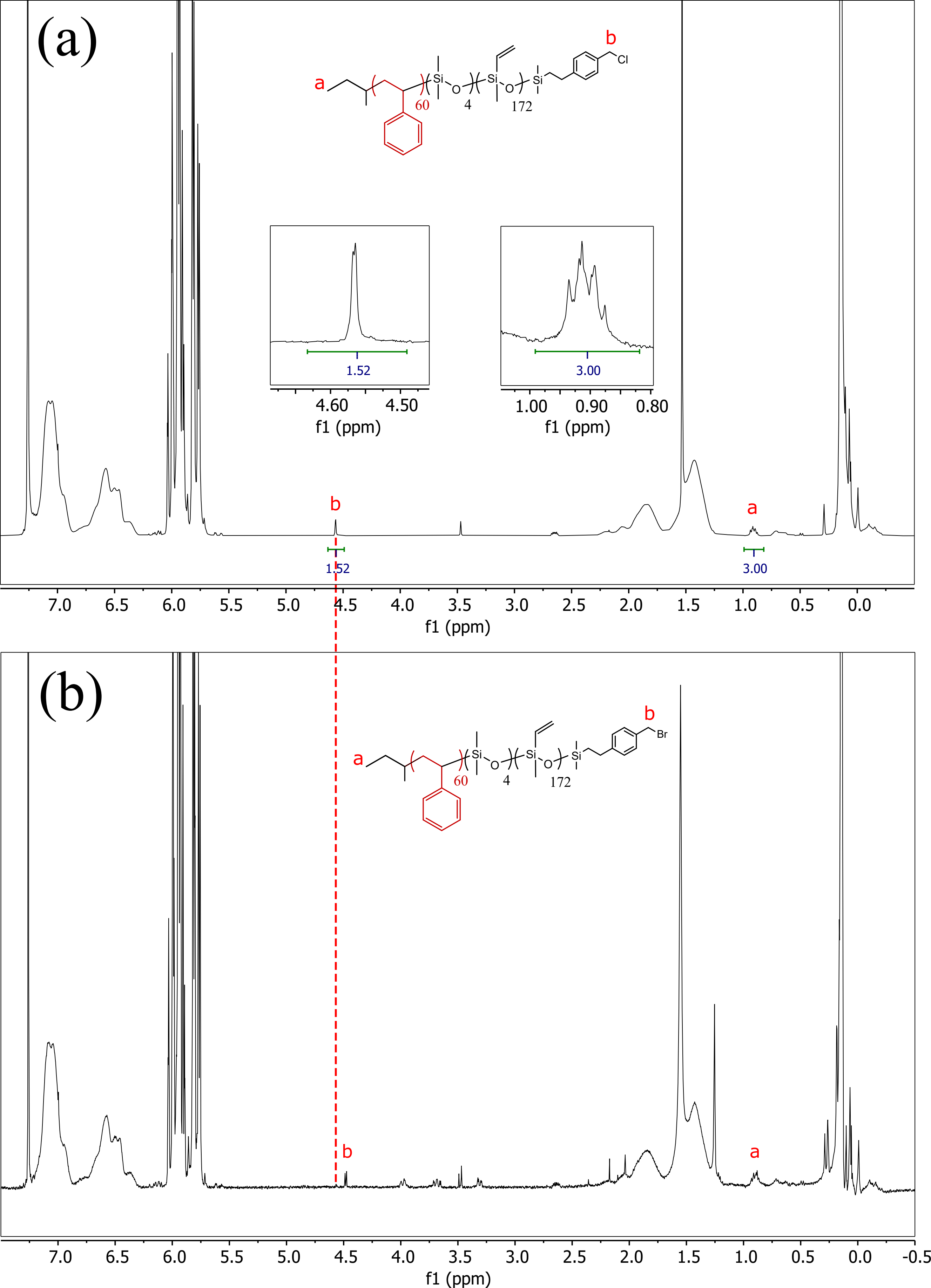

(a)
a
b
Si
O
4
Si
O
172
Si
Cl
60
1.52
4.60
4.50
f1 (ppm)
3.00
1.00
0.90
0.80
f1 (ppm)
b
a
1.52
3.00
7.0 6.5 6.0 5.5 5.0 4.5 4.0 3.5 3.0 2.5 2.0 1.5 1.0 0.5 0.0
f1 (ppm)
(b)
a
b
Br
60
4
172
b
a
7.0 6.5 6.0 5.5 5.0 4.5 4.0 3.5 3.0 2.5 2.0 1.5 1.0 0.5 0.0 -0.5
f1 (ppm)
